# Development of a Human 3D Immune‐Competent Neurovascular Model Enabling Time‐Resolved Monitoring of Neuroinflammatory Dynamics and Neuroimmune Interactions

**DOI:** 10.1002/advs.75461

**Published:** 2026-04-29

**Authors:** Pavlo Gordiichuk, Jing Bai, Olurotimi A. Bolonduro, Andrew M. Silverman, Robert Madison Green, Jason H. Lasser, Paul W. Fleming, Lishomwa C. Ndhlovu, James F. Saunders, Dmitry Shvartsman

**Affiliations:** ^1^ Triton Systems, Inc. Chelmsford Massachusetts USA; ^2^ Division of Infectious Diseases Department of Medicine Weill Cornell Medicine New York New York USA

**Keywords:** blood–brain barrier, microfluidics, microphysiological systems, neuroinflammation, organoids, TEER

## Abstract

Neuroinflammation disrupts the blood–brain barrier (BBB) and drives neurological disease, but current in vitro MPS and animal models fail to capture its dynamic, human‐specific immune complexity. Here, we introduce a 3D human immune‐competent BBB (3D‐HIC‐BBB) platform that reconstitutes the neurovascular unit (NVU) by integrating primary human brain microvascular endothelial cells, astrocytes, brain vascular pericytes, human microglia, and dopaminergic neurospheroids with embedded transepithelial electrical resistance (TEER) microsensors for continuous functional monitoring. Our platform design enables high‐resolution of inflammatory NVU dynamics that are inaccessible to conventional endpoint assays. Using controlled exposure to tumor necrosis factor‐α (TNF‐α) and lipopolysaccharide (LPS), two distinct triggers of immune responses, we observed stimulus‐specific neuroinflammatory responses with distinct temporal and functional signatures with the resistance full width at half maximum (FWHM) for LPS = 15.8 h and TNF‐α  = 4.3 h. TNF‐α exposure elicited a lesser (Δ345 Ω·cm^2^) but faster drop in resistance and largely faster and reversible recovery than LPS, consistent with transient cytokine‐driven disruption. In contrast, LPS induced a delayed yet more severe (Δ560 Ω·cm^2^) loss. The 3D‐HIC‐BBB platform distinguishes endotoxin‐ from cytokine‐driven NVU dysfunction and reveals differences in cytokine profiles. It provides a biologically relevant, predictive model for studying neuroinflammation and evaluating anti‐inflammatory therapies.

## Graphical abstract

Neuroinflammation alters blood–brain barrier integrity and contributes to neurological disorders, yet existing models lack human immune complexity. This study presents a 3D, immune‐responsive platform that reconstructs key neurovascular components and enables real‐time monitoring. Distinct inflammatory triggers produce unique temporal and functional responses, offering improved insight into barrier dysfunction and a more predictive system for therapeutic evaluation.

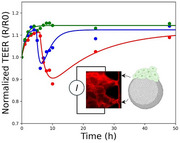

## 1 Introduction

Neuroinflammation is a complex and tightly regulated immune response within the central nervous system (CNS) that arises in response to infection, trauma, ischemia, or neurodegenerative pathology [1, 2] and it is primarily mediated by resident glial cells—microglia and astrocytes [3, 4]. Microglia serve as the innate immune sentinels of the CNS and rapidly activate upon sensing injury‐ or pathogen‐associated signals, releasing pro‐inflammatory cytokines such as tumor necrosis TNF‐α, interleukin‐1β (IL‐1β), and interleukin‐6 (IL‐6), as well as reactive oxygen and nitrogen species [5, 6]. In response, astrocytes undergo reactive astrogliosis, amplifying inflammatory signaling, altering metabolic support, and modulating BBB permeability [7, 8].

Despite extensive investigation, the spatiotemporal coupling between NVUs and microglial activation, cytokine signaling, and BBB dysfunction remains incompletely understood, largely due to limitations of existing experimental models, which cannot capture the kinetics of individual inflammatory processes [9]. Thus, there is a need for the development of human 3D immune‐competent neurovascular models combined with tools capable of measurement of BBB functions, not merely as a passive physical barrier but as an active immunological interface that integrates inflammatory cues from both the CNS parenchyma and the peripheral circulation [10]. In this context, the BBB physical function is heavily relied on the composed of brain‐microvascular endothelial cells supported by a capillary basement membrane, embedded pericytes, and astrocytic end‐feet, which together regulate molecular transport, immune surveillance, and vascular homeostasis [3, 11]. By monitoring time‐dependent remodeling of endothelial tight junctions and cytoskeletal organization, key determinants of BBB integrity that are highly sensitive to immune activation [12, 13], we used longitudinal sensing to distinguish inflammatory models based on the rate and magnitude of junctional disruption and the extent of self‐recovery in the context of the NVU (e.g., responses to inflammatory cytokines and pathogen‐associated molecular patterns).

Therefore, a central challenge in neuroinflammation research is distinguishing transient, potentially reversible inflammation from sustained, self‐amplifying inflammatory states that drive chronic BBB disruption and are reinforced by NVU. This distinction is exemplified by commonly used inflammatory stimuli such as TNF‐α and LPS, which engage fundamentally distinct signaling mechanisms [14]. TNF‐α primarily signals through TNF receptors (TNFR1 and TNFR2) expressed on endothelial cells, astrocytes, and microglia, activating NF‐κB and MAPK pathways that transiently disrupt tight junction organization and increase paracellular permeability [15, 16]. Importantly, TNF‐α‐mediated barrier dysfunction is often reversible, as feedback mechanisms promote junctional reassembly and restoration of endothelial integrity following resolution of the stimulus [13].

In contrast, LPS activates Toll‐like receptor 4 (TLR4), which is highly expressed on NVU and endothelial cells, initiating both MyD88‐dependent and TRIF‐dependent signaling cascades [17, 18]. This dual‐pathway activation results in prolonged NF‐κB signaling, interferon responses, and amplified cytokine and chemokine release [19]. In immune‐competent neurovascular systems, LPS stimulation drives secondary waves of microglia‐mediated inflammation that reinforce endothelial dysfunction, promote sustained tight junction degradation, and suppress barrier recovery [20]. Consequently, LPS exposure is typically associated with persistent BBB breakdown rather than transient permeability changes [21].

Many in vivo and in vitro models employed to study neuroinflammation present important limitations and are incapable of capturing transient or sustained cells damage. For example, animal models often fail to recapitulate human‐specific immune signaling and BBB regulation, contributing to poor translational predictivity in CNS responses to different inflammation models or testing drug formulations [22, 23]. Respectively, conventional in vitro BBB models, including transwell‐based systems, rely largely on 2D endothelial monolayers and lack NVU as well as spatial 3D organization of the brain cells mimicking human brain structures and interaction scenarios [9].

Recent advances in organ‐on‐chip and MPS technologies have enabled the integration of TEER electrodes directly into microfluidic platforms, allowing continuous, noninvasive monitoring of the endothelial cells barrier [24, 25]. It has been demonstrated that the integrated‐electrode TEER systems reduce handling artifacts and improve temporal resolution compared to conventional “chopstick” approaches and allows development of high‐throughput platforms enable parallelized time‐lapse measurements under controlled flow conditions [26]. However, most existing TEER‐enabled BBB chips are optimized for simplified endothelial architectures and lack complex 3D vascular organization, immune components, and, critically, microglia, which are critical for replication of human‐competent neuroinflammation kinetics [27, 28, 29]. Moreover, differences in electrode geometry and chip architecture complicate cross‐platform comparisons of absolute TEER values, underscoring the importance of analyzing within‐platform dynamic signatures—such as resistance decline and recovery profiles—rather than relying solely on endpoint measurements [30].

Separately, 3D in vitro neurovascular models, including vascularized brain organoids and induced pluripotent stem cell–derived spheroids, have improved physiological relevance by enhancing tight junction expression and increasing TEER values [31, 32]. However, these systems often lack human‐scale vascular dimensions as limited to miniaturized micro vessels limiting their capacity to model inflammation‐driven BBB dysfunction and accurate time‐dependent responses of inflammation models [33].

Here, we present the development and characterization of immune‐competent, 3D human‐integrated CNS blood–brain barrier (e.g., 3D‐HIC‐BBB) MPS that combine neurovascular architecture with resident immune cells/neurons and integrated, time‐dependent TEER sensing. In contrast to prior models focused primarily on BBB barrier function, our platform is designed to capture BBB dynamics in conjunction with NVUs and neurospheroids, enabling discrimination among inflammatory stimuli and differentiation between transient and sustained barrier dysfunctions. These capabilities are broadly applicable to neurological diseases in which neuroinflammation and BBB disruption are central pathogenic drivers. These models are of paramount importance for a wide range of studies in which BBB permeability is altered by inflammatory diseases, particularly those in which barrier dysfunction plays a central pathogenic role leading to chronic diseases. In neurodegenerative disorders such as Alzheimer's and Parkinson's diseases, chronic microglial activation and sustained cytokine signaling are associated with progressive BBB leakage and impaired clearance of neurotoxic protein aggregates [12, 34]. In multiple sclerosis, immune‐mediated BBB disruption facilitates leukocyte infiltration and demyelination, with barrier dysfunction fluctuating across relapse–remission cycles [35]. Acute CNS injuries, including ischemic stroke and traumatic brain injury, involve rapid cytokine‐driven BBB breakdown followed by prolonged secondary inflammation that contributes to delayed neurological damage [36].

Moreover, this system enables investigation of infectious and post‐infectious neurological conditions. Viral infections such as HIV and SARS‐CoV‐2 infection, induce multi‐phase neuroinflammatory responses that compromise BBB integrity through both direct viral effects and microglia‐mediated cytokine cascades [37]. Neuroinflammation has also been implicated in neuropsychiatric and neurodevelopmental disorders, where subtle but persistent alterations in BBB function and immune signaling may contribute to disease progression [38, 39]. By enabling continuous, quantitative discrimination between transient and sustained inflammatory barrier dysfunction, immune‐competent 3D BBB MPS platforms provide a versatile framework for mechanistic investigation and therapeutic evaluation across a wide spectrum of neuroinflammation‐driven CNS diseases.

In summary, we demonstrated that 3D‐HIC‐BBB MPS replicates inflammatory conditions (e.g., LPS‐ or TNF‐α–induced inflammation) with responses that closely replicate human physiology responses, as evidenced by the extent of endothelial junction disruption and the kinetics of tissue functional recovery measured through time‐dependent TEER changes. Our assay analysis confirmed that distinct inflammatory stimuli elicit unique response patterns, and that continuous TEER measurements effectively capture response profiles of the 3D‐HIC‐BBB chips. Our work intended to replicate the previously established 3D human‐relevant BBB neuroinflammation models and advances them by incorporating 3D vascular structures into 3D brain tissue containing microglia and neurospheroids [40, 41, 42]. This work aligns with and supports the growing adoption of New Approach Methodologies (NAMs) approved by the FDA that enhance human relevance while reducing reliance on animal testing and align with new regulatory mandates in the US and EU [43, 44]. Our findings stay aligned with MPSs that integrate physiologically relevant cellular complexity with continuous, quantitative functional readouts as promising NAMs for defined contexts of use in nonclinical drug development [45].

## 2 Results

### 2.1 Design and Architecture of the Microphysiological NVU Platform With Embedded TEER Sensor

Our study shows that, in the developed human 3D immune‐competent neurovascular tissue models (e.g., 3D‐HIC‐BBB), TEER exhibits both amplitude drops and subsequent recoveries following exposure to inflammatory stimuli. These responses yield stimulus‐specific sensing metrics, including the magnitude of TEER decrease and recovery (e.g., FWHM), as well as decay and rise times that predict complete or incomplete tissue recovery. These experiments are consistent with reported measurements [46, 47], while suggesting that the 3D‐HIC‐BBB MPS provides higher sensitivity to inflammation‐induced responses and kinetics. In contrast to the inflammation conditions, we observed an increase in TEER in control batches, which may reflect viscoelastic changes in the cells due to temperature shifts or measurement‐related stress (e.g., measurements were conducted at room temperature).

In contrast to previous studies, where TEER measurements were used for barrier integrity across simpler BBB tissues [26, 29, 30, 48], our TEER measurements in the 3D‐HIC‐BBB model demonstrated high sensitivity to inflammation dynamics, such as time‐dependent disruption in junctional integrity followed by barrier recovery. While TEER measured in the 3D vascular structure of the 3D‐HIC‐BBB MPS depends on vessel density, as reflected in variability in TEER amplitude across devices, it has demonstrated its value in time‐dependent measurements for understanding inflammation and healing. The similar TEER changes were seen in complex systems such as printed, sliced, or live human tissue, as a label‐free physical parameter [49, 50, 51, 52, 53, 54, 55, 56]. Therefore, such electrical sensing strategies are readily scalable and compatible with chip‐based arrays, which support development and characterization of 3D‐HIC‐BBB rays enabling high‐throughput screening and rapid assessment of BBB function in laboratories setting as NAMs.

**FIGURE 1:**
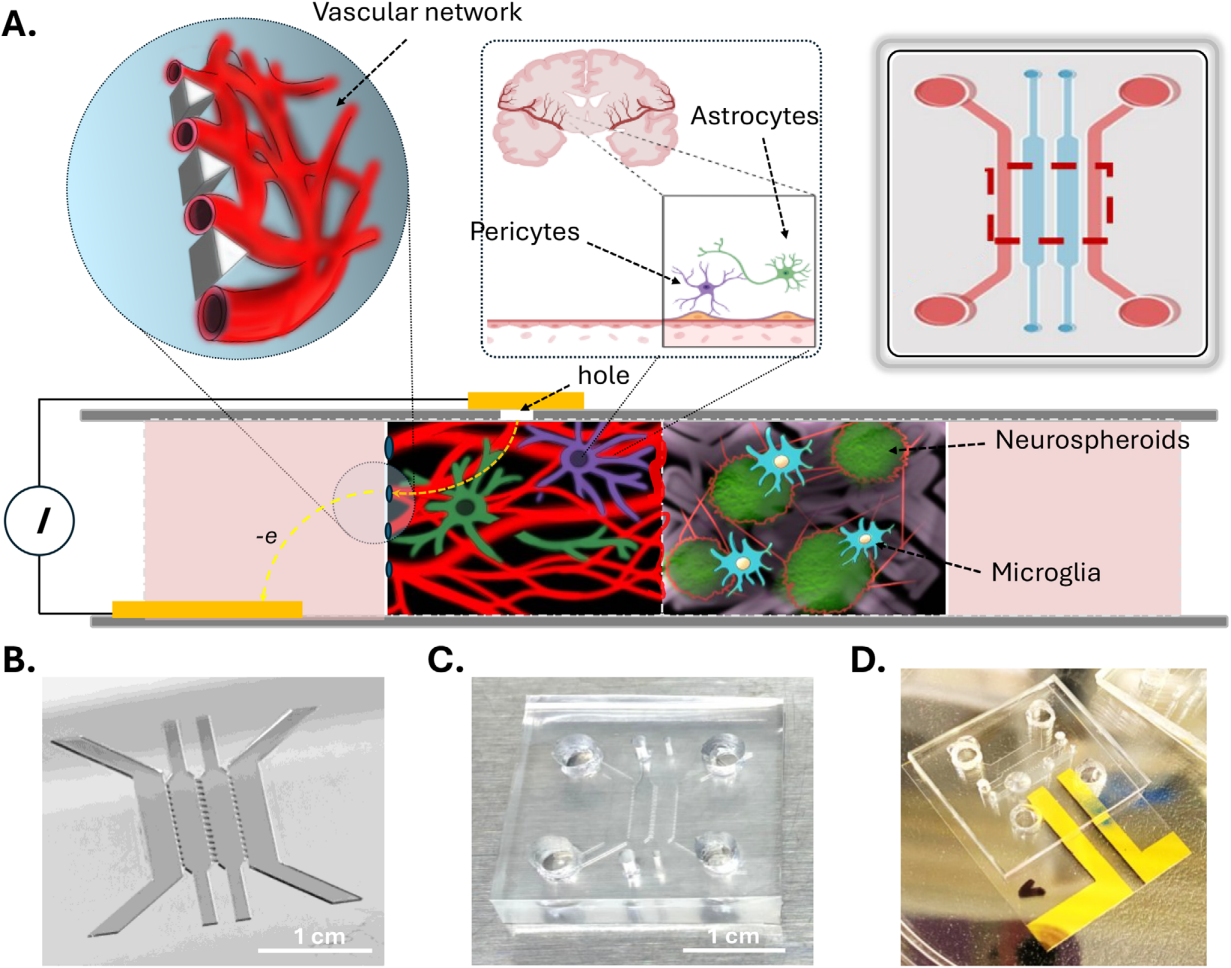
Overview of the 3D BBB–neurovascular co‐culture workflow and device platform. (A) Representative image of the BBB microvessel network with CD31^+^ brain endothelial vasculature (red). Image of the device showing two gel channels (in blue: #2, and #3 from left to right) and two perfusion media channels (in red: #1 and #4 from left to right). The bottom image shows a cross‐section of the device, where the BBB–neuron–immune co‐culture is developed. Top and bottom gold (Au) electrodes are used for TEER measurements. In our nutrients supply channel (#1) we used glass with prefabricates gold contacts. In our microfluidic design, the vascular channel (#2) was established first, and we added holes in PDMS for TEER top contact, while channel #3 was left empty from cells until the vasculature had formed. Subsequently, channel #3 was established with the NVU. (B) A Si template fabricated by dry etching for the microfluidic device. (C) Picture of the PDMS device fabricated from templated molds, incorporating holes created using punches. (D) Microfluidic device integrated with TEER electrodes for barrier measurement. See detailed geometrical parameters on Figure S1. We developed four parallel microfluidic channels, in which the inner channels (#2 and #3) were used for the vasculature and NVU, respectively. In contrast, the outer channels (#1 and #4) were used to supply nutrients and facilitate TEER measurements. Thus, the bottom glass substrate had gold contacts that were exposed only to channel #1. In contrast, we fabricated holes in the PDMS above channel #2 on the top to facilitate the electrical connection from the top Au electrodes to the vascular (see Figure S5).

We designed microfluidic chips that incorporate an NVU, including both the BBB and neurons and microglia, with integrated TEER sensors to enable time‐dependent, noninvasive monitoring of BBB integrity. Figure 1A shows the engineered vasculature grown on‐chip and its interaction with the NVU across separated compartments, recapitulating key aspects of brain physiology. In addition, because reliable TEER measurements require the formation of a continuous, single‐layer 3D BBB structure, we controlled the channel height (250 µm) in the microfluidic device to ensure uniform barrier formation and stable TEER readouts (Figure 1A). To achieve this, we designed and fabricated PDMS devices with four microchannels molded from silicon masters (see Methods; Figure 1B,C). We designed 2 mm channels in PDMS using silicon‐etched molds (see Methods), where the middle portions of the channels were partially separated by rows of trapezoidal pillars with dimensions of 0.2 mm × 0.4 mm (see Figure S1). Each trapezoidal row contained 15 features in the silicon master, which were used to separate cells during the infusion process. The two middle rows contain a double row of pillars to provide mechanical reinforcement for the channels. The height of the channels was designed to be 250 µm, and the PDMS thickness ranged from 3 to 5 mm. The two central, side‐by‐side channels (blue) were used to culture the vasculature and brain tissue, respectively, and were separated by embedded triangular PDMS stoppers. The two outer channels (red) served as media reservoirs to support tissue growth and were similarly isolated using stoppers.

For TEER measurements, gold contact pads were patterned on a glass substrate (see Methods) and used to interface with the PDMS device from below prior to initiating tissue culture. Thus, the bottom glass substrate had gold contacts that were exposed only to channel #1. In contrast, we fabricated holes in the PDMS above channel #2 on the top to facilitate the electrical connection from the top Au electrodes to the vascular channel. In detail, a second glass substrate with gold electrodes was placed on top of the PDMS device on the day TEER measurements were performed (Figure 1D). The electrodes were arranged such that current passed between the vasculature and the adjacent channel through the formed, perfused vascular walls. In this configuration, the electrical resistance of the vascular structures was measured between the top and bottom electrodes across the PDMS (∼1 mm). Importantly, measurements were made through prefabricated access holes in the PDMS, so the current path between contacts traversed the perfused vasculature integrated with the NVU (Figure 1A).

To optimize the BBB cell configurations, we first optimized vascular network formation by comparing endothelial cell (EC) sources and stromal cell ratios (Figure S2). Compared to iPSC‐derived ECs at day 7, HBMECs formed more continuous and interconnected vascular networks, whereas iPSC‐EC conditions showed regions with incomplete vessel connections, likely due to differences in endothelial maturity and junctional organization. In addition, varying astrocyte (AC) and pericyte (PC) ratios affected network morphology. We applied a higher endothelial‐to‐pericyte ratio in our co‐cultures to ensure the formation of well‐defined 3D vascular structures; this ratio could be reduced in future experiments [57, 58]. We designed four groups: (a) EC:PC:AC = 10:1:2; (b) Half AC—EC:PC:AC = 10:1:1; (c) Half PC—EC:PC:AC = 20:1:4; and (d) Half AC + PC—EC:PC:AC = 20:1:2. These results indicate that both AC and PC—and their relative proportions—are critical for optimal BBB formation. In particular, reduced PC content can compromise vessel stability and maturation, while AC and PC together synergistically support robust BBB‐like vascular development. Based on these findings, we used the group EC:PC:AC ratio (10:1:2) for subsequent experiments (final concentration is: EC = 5 × 10^6^ cells/mL, PC: 5 × 10^5^ cells/mL, and AC: 1 × 10^6^ cells/mL). In our microfluidic channels, the co‐culture of endothelial cells with pericytes promoted the formation of stable, capillary‐like networks. The pericytes provided structural support and secreted pro‐angiogenic factors, such as VEGF and angiopoietin‐1, which synergize with endothelial signaling to enhance sprouting, lumen formation, and vessel maturation. These observations are consistent with previous studies showing that pericyte‐mediated signaling supports network self‐organization and stabilization in engineered microvascular systems [59, 60]. Based on these observations and literature support, HBMECs and the control AC/PC ratio were selected for subsequent experiments [61].

**FIGURE 2:**
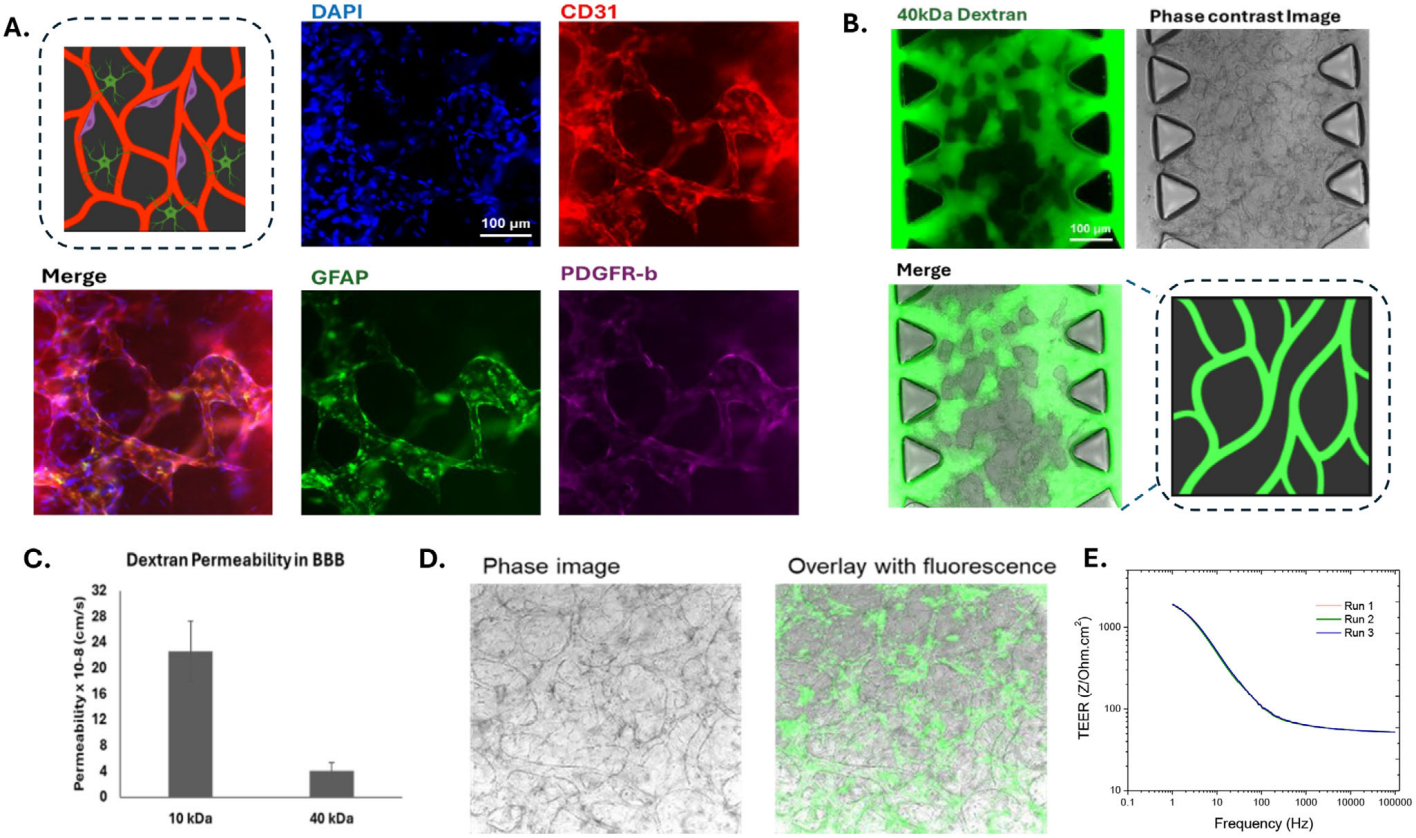
Characterization and functional assessment of the 3D self‐assembled BBB microvasculature. (A) Immunostaining of the vascular network showing endothelial cells (CD31), astrocytes (GFAP), and pericytes (PDGFR‐β), with DAPI marking nuclei. Merged images demonstrate coordinated organization of BBB‐associated cell types. (B) Perfusion of 40 kDa fluorescent dextran through the microvascular network confirms lumen continuity and successful barrier formation. The system's microvasculature consists of a single endothelial cell layer forming a perfusable lumen, therefore, the relative time‐dependent TEER measurements mainly reflect transport across this single barrier rather than multiple cell layers. (C) Quantification of dextran permeability analysis. Data are presented as mean ± SEM (*n* = 3 independent chips). This experiment was performed to assess size‐dependent permeability as a functional validation of barrier selectivity. (D) Brightfield and fluorescent overlays show the morphology of GFP‐VECadherin BMVEC vessels. (E) A typical TEER measurement over the same device showed stability of runs (Figure S5). To ensure that ion flow occurred solely through the vasculature, the bottom TEER electrode contacted channel #1 while the top electrode was positioned above channel #2. Electrical measurements were therefore established only between channels #1 and #2, preventing direct electrical influence on the NVU channel. This electrode configuration and tissue architecture ensured that communication between channels #2 and #3 occurred exclusively through biological interactions, enabling measurement of time‐dependent disruption and recovery of the 3D vascular structures [25, 29, 67, 68, 69].

Next, we performed immunofluorescence (IF) staining on day 7 (Figure 2A). The IF images provide strong evidence for the presence and organization of key BBB cell types within the co‐culture. Co‐localization of endothelial cells (CD31), astrocytes (GFAP), and pericytes (PDGFR‐β) supports the formation of the BBB structure. We then evaluated BBB functionality by quantifying dextran permeability using 10 kDa GFP‐conjugated dextran and 40 kDa Texas Red–conjugated dextran and compared the results with reported in vivo BBB permeability ranges (Figures S3 and S4). On day 7, our 3D BBB model exhibited physiologically relevant permeability values comparable to those measured in animal models: for 40 kDa dextran, 1.4–1.9 × 10^−^^7^ cm/s reported in rats [62] vs. 4.2 × 10^−^^8^ cm/s in our model; for 10 kDa dextran, 3.1 × 10^−^^7^ cm/s reported in rat brain capillaries [63] vs. 2.3 × 10^−^^7^ cm/s in our model (Figure 2B,C). Our work demonstrated perfusion regimes similar to those reported in the literature by growing tissue and the medium in the reservoirs was changed and leveled every other day to maintain interstitial flow [64, 65, 66].

A single‐layer vascular network was successfully established. The network showed no gaps at the interface between the tissue chamber and the main vessel channel, indicating a well‐formed, sealed endothelial barrier (Figure 2A). Importantly, an open and perfusable connection to the main channel was maintained, which is critical for accurate TEER measurements by ensuring stable electrode access and uninterrupted barrier continuity. To enable noninvasive quantification of vessel area—critical for accurate TEER normalization—we transitioned to GFP‐labeled VE‐cadherin human brain microvascular endothelial cells (HBMECs), which allow direct visualization and tracking of vessel structures during live imaging. These cells are identical to the previously used HBMECs, except for the addition of a fluorescent reporter that enables live imaging of vascular structures (Figure 2B,C). Using brightfield and I imaging, we observed the formation of a self‐assembled 3D vasculature within the microfluidic device (Figure 2D). Strong GFP expression confirmed robust vascular network formation, with an average vessel diameter of 40–100 µm. Importantly, these GFP‐labeled cells exhibited permeability comparable to the parental, non‐labeled HBMECs (∼5.6 × 10^−^^8^ vs. 4.2 × 10^−^^8^ cm/s, respectively).

After completing the optical validations and prior to the inflammation experiments, we performed TEER measurements on four fabricated devices (see Methods) to assess sensor sensitivity across the engineered tissue (Figure 2E). The resulting Bode plots showed high stability and reproducibility, with <1% variability within the same device and <10% variability across different devices/samples. The between‐device variability likely reflects device‐to‐device fabrication differences as well as sample‐specific tissue architecture. Overall, the measured TEER resistances fell within the range reported for comparable BBB models [70]. Similarly to 2D endothelial cell cultures, the 3D vascular structures established here consist of ECs forming a continuous monolayer that acts as an electrical barrier allowing current to flow via vessels perfused vessels walls. Although geometry and 3D architecture influence the overall barrier area [71, 72, 73], the integrity of the vascular structures during inflammatory stimulation exhibited patterns of disruption and recovery comparable to those observed in analogous 2D systems. Our TEER measurements are consistent with previous reports on 3D vascular networks [74, 75, 76] and are further influenced by biological interactions with the NVU.

### 2.2 Establishment of Multicellular NVU Components

#### 2.2.1 Development of an In Vitro Neurovascular Unit Co‐Culture Model

Dopaminergic neurons were selected as a representative neuronal subtype to assess downstream effects of infection‐related neurological disorders, as well as to capture their selective vulnerability and functional relevance in neurodegenerative processes. To ensure reproducibility and translational consistency, these neurons were derived from ATCC neural progenitor cells (NPCs), which provide a standardized, well‐characterized source with robust differentiation potential and reduced batch‐to‐batch variability. To optimize spheroid formation from neural NPCs, we evaluated two initial seeding densities: 2000 cells/well (ATCC‐recommended) and 4000 cells/well. Brightfield images were acquired on Day 1 (1‐day post‐seeding) and Day 8 (1 day after initiating dopaminergic differentiation) (Figure S6). On Day 1, both conditions produced compact spheroids with minimal structural differences. By Day 8, no significant size differences were observed between the two groups; however, spheroids seeded at 2000 cells/well appeared more uniform and well‐formed, with fewer peripheral cells and more defined edges. Although spheroids seeded at 4000 cells/well exhibited slightly denser cores, the 2000 cells/well condition provided better overall consistency. These results suggest that the lower seeding density is more suitable for generating reproducible spheroids for downstream differentiation and MPS integration. Therefore, we used 2000 cells/well for subsequent experiments.

**FIGURE 3:**
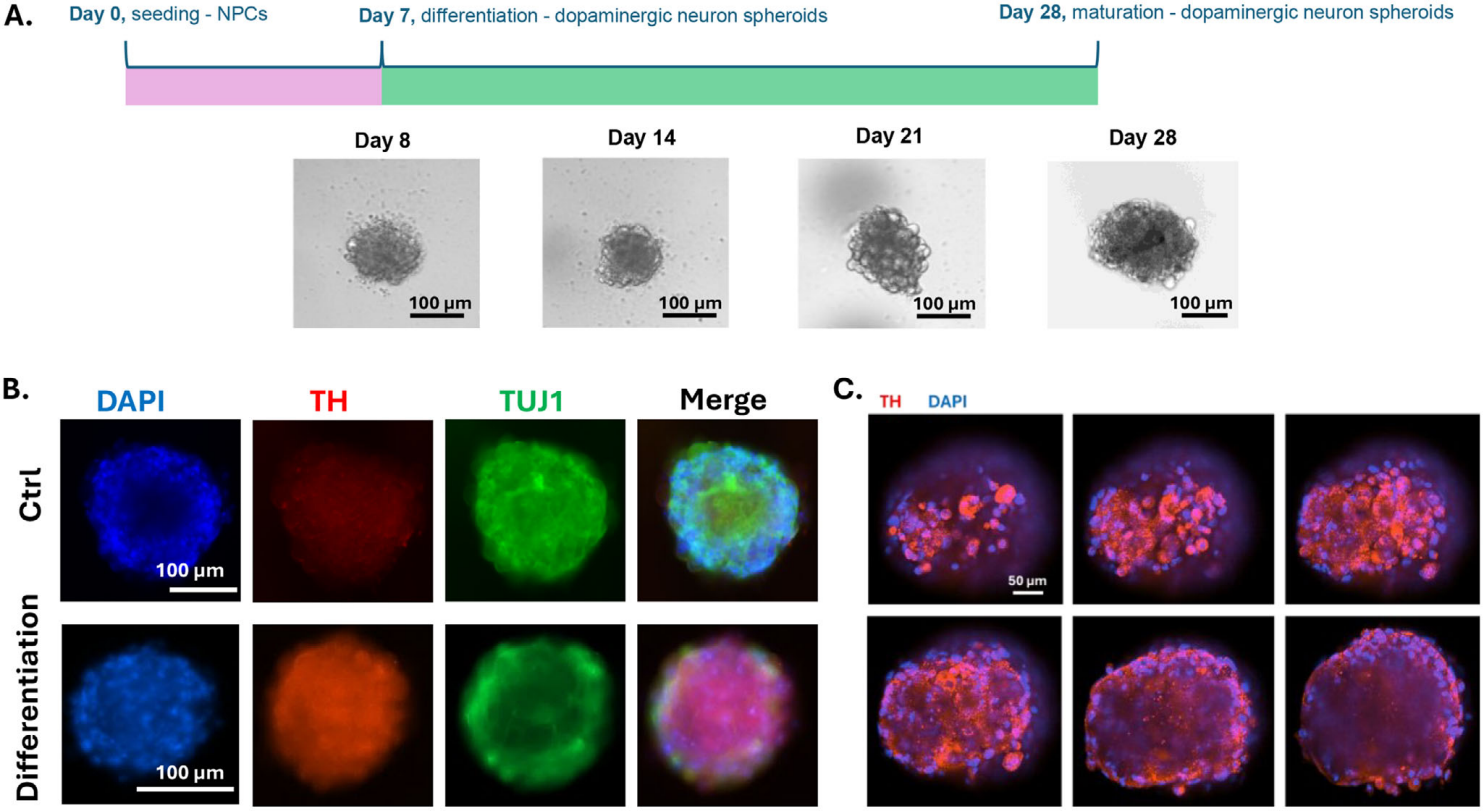
Characterization of dopaminergic neurospheroids. (A) Differentiation and maturation timeline of dopaminergic neuron spheroids derived from NPCs. Representative brightfield images show progressive spheroid morphological development from early aggregation to mature dopaminergic neuron spheroids. Differentiation condition (bottom row) promotes dopaminergic specification, increasing TH expression ∼2.4‐fold (1.39/0.57) relative to TUJ1 without altering overall cell density, accompanied by spatial reorganization of neuronal subtypes within organoids. (B) IF of Day 28 dopaminergic neurospheroids showing DAPI, TH, and TUJ1 expression, confirming neuronal enrichment and dopaminergic phenotype. The IF images were statistically analyzed (see Figures S8 and S9). (C) Cross‐sectional images of neurospheroids stained for TH and DAPI demonstrating dense dopaminergic neuron populations.

Next, to evaluate morphological changes during dopaminergic differentiation, spheroids were imaged on Day 8, Day 14, Day 21, and Day 28 of culture, corresponding to Days 1, 7, 14, and 21 post‐differentiation, respectively. (Figure 3A). These observations suggest that the NPC phenotype remains detectable through Day 21 of differentiation. By Day 28 (equivalent to Day 21 of differentiation), spheroids from both groups were fixed and stained for neural progenitor cell markers (PAX6 and Nestin). The control (Ctrl) group consisted of spheroids without dopaminergic induction, while the Differentiation group received dopaminergic induction. IF imaging showed strong expression of both PAX6 and Nestin in the Ctrl group, confirming maintenance of NPC identity. In the Differentiation group, PAX6 expression decreased, while Nestin expression showed some variability (Figure S7). We interpret this as expected, as differentiation protocols often yield mixed populations containing both dopaminergic neurons and residual NPCs, and complete downregulation of Nestin may require longer culture (>28–35 days of differentiation).

Next, we evaluated dopaminergic neuronal differentiation in NPC‐derived spheroids cultured under identical low‐adhesion conditions, using the same two experimental groups (Figure 3B). IF staining was performed for tyrosine hydroxylase (TH; red), a key enzyme in dopamine synthesis, and TUJ1 (βIII‐tubulin; green), a pan‐neuronal marker, with DAPI nuclear counterstaining (blue). In the Differentiation group, robust expression of both TH and TUJ1 was observed, indicating successful induction of dopaminergic neuron‐like cells. In contrast, the Ctrl group exhibited minimal TH expression. We also summarized sectional confocal views of neurospheroids from Day 28 cultures; Figure 3C shows an enlarged view highlighting TH expression throughout the spheroid. Together, these results demonstrate that the applied differentiation protocol effectively promotes dopaminergic lineage commitment, with TH+ neuron formation evident by Day 21 of differentiation.

We evaluated the survival of HMC‐3 human microglial cells (ATCC, CRL‐3304) under non‐adherent conditions. Brightfield imaging (Figure S10A) and fluorescence imaging revealed that the majority of cells remained viable (Figure S10B), with only a few red‐stained dead cells observed. These results suggest that HMC‐3 microglia can survive under low‐adhesion conditions, supporting the feasibility of future experiments involving microglia–neuron spheroid fusion or immune‐modulated neurosphere models. The subsequent IF panels confirm that the microglial cells express both IBA1 and CD11b, indicating preserved identity and potential activation status after 48 h in suspension (Figure S10C).

**FIGURE 4:**
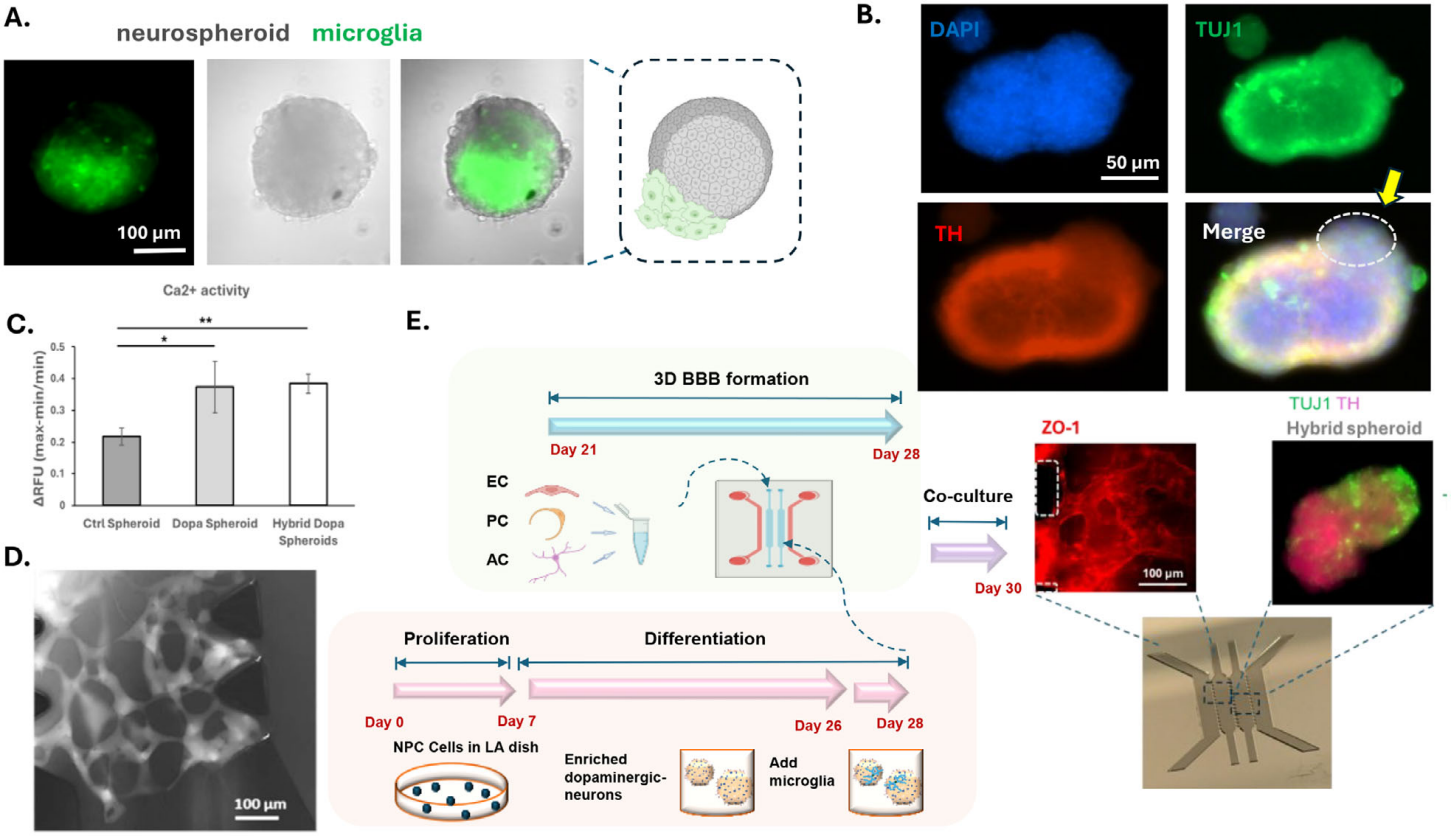
Characterization of hybrid neurospheroids with microglia. (A) Formation of hybrid neurospheroids by introducing microglia (green). Brightfield and fluorescence images show successful microglia–neuron integration. (B) IF of Day 28 hybrid neurospheroids showing DAPI, TH, and TUJ1 expression. (C) Calcium activities comparison of NPC‐only spheroids, dopaminergic spheroids, and hybrid dopaminergic–microglia spheroids. The merged image confirms microglial association and localization at the periphery and partially within the spheroid (yellow arrow). (D) 40 kDa GFP dextran perfusion showing vessel openings on only one side, with the opposite, brain‐facing side sealed. Schematic of 3D neurovascular model formation. (E) Integration of hybrid spheroids into the central chamber of the BBB device to establish a neurovascular co‐culture. Representative images of BBB networks (ZO‐1), hybrid spheroids (Tuj1/TH), and device architecture. Hybrid spheroids were embedded within a hydrogel matrix inside the microfluidic channels. This configuration provides mechanical support, maintains spheroid position during perfusion, and allows microglia to migrate along the spheroid periphery or into the surrounding gel.

To establish an MPS model considering of neuroimmune interaction, we successfully generated hybrid neurospheroids by co‐culturing the dopaminergic neurospheroids with microglia in suspension. As shown in Figure 4A,B, IF staining confirmed neuronal identity through TUJ1 expression and dopaminergic lineage via TH positivity. A discontinuity at the spheroid edge (indicated by the yellow arrow) suggests the physical presence and integration of microglia into the neuronal structure.

Live imaging of the GFP‐labeled microglia (CellTracker Fluorescent Probes, green, CMFDA) revealed their localization around and partially within the neurospheroids, further supporting microglial association with neuronal tissue. These findings demonstrate the successful formation of hybrid spheroids that can be used to model microglia–neuron interactions in a controlled 3D environment, suitable for downstream studies of neuroinflammation.

To assess dopaminergic neurons functionality, calcium activity was measured across three spheroid types using a fluorescence‐based Ca^2^^+^ assay, dopaminergic spheroids exhibited significantly higher calcium activity (∼0.37 ΔRFU) compared to control spheroids (∼0.22 ΔRFU), indicating enhanced functional maturation and excitability following dopaminergic differentiation. Notably, hybrid dopaminergic spheroids (co‐cultured with microglia) showed comparable calcium activity (∼0.38 ΔRFU) to the dopaminergic‐only group, suggesting that the presence of microglia does not impair neuronal excitability. These findings support successful dopaminergic neuron differentiation and suggest the hybrid system remains functionally active (Figure 4C).

We observed the formation of a BBB model with asymmetric vessel perfusion, mimicking selective transport across the BBB into the brain parenchyma (Figure 4D; Figure S11). Detailed methods description can be found in Figure S11. We also evaluated the effects of culture media composition (BBB vs. neurospheroid media) on neuronal identity and calcium activity in dopaminergic neurospheroids (Figure S12A). We also quantified Ca^2^^+^ activity in spheroids cultured in three media conditions: EC media, dopaminergic media, and a 1:1 mixture of the two. Calcium activity was assessed using Fluo‐4 dye and expressed as ΔRFU (max–min per minute). No statistically significant differences in Ca^2^^+^ activity were detected among the three media conditions (ΔRFU in EC media: 0.27 ± 0.12, in Dopa media: 0.37 ± 0.03, in 1:1 Dopa + EC media: 0.35 ± 0.04). Notably, the EC media group exhibited substantially greater variability compared to the other two conditions. The lack of significance is likely attributable to this high intra‐group variability combined with the limited sample size. These results suggest that while EC media supports neuronal viability to some extent, dopaminergic‐specific media—or a blended formulation—is more effective for sustaining neuronal function (Figure S12B).

Together, these findings demonstrate that media composition strongly influences neuronal function and excitability in dopaminergic neurospheroids in co‐culture. We used a 1:1 mixture of the two media conditions for the neuronal chamber in the co‐culture. In the co‐culture experimental setup (Figure 4E), approximately 10 hybrid neurospheroids (∼20 000 neural cells per channel) were loaded into the central tissue chamber of each device. Dopaminergic neuronal traits were confirmed by TUJ1 (green) and TH (magenta). Media composition was compartment‐specific: BBB endothelial channels were maintained in EC growth media, while the neuronal channels received a 1:1 mixture of EC media and dopaminergic differentiation media to support both vascular and neuronal components. While the function of a typical 3D vascular BBB is commonly characterized using tight junction proteins such as ZO‐1 (Invitrogen), occludin, and claudin‐5 [77], in this study, we limited our analysis to ZO‐1. The endothelial junction protein ZO‐1 (red) indicates tight junction formation and maintenance of BBB integrity during co‐culture. These results also support the formation of a structurally organized and functionally restrictive barrier. Co‐culture conditions were sustained for 48 h prior to induction of any inflammatory stimulation, allowing functional integration and stabilization of the neurovascular interface.

#### 2.2.2 Study Cellular Behavior in an Induced Inflammatory Microenvironment

We optimized a protocol to induce inflammatory conditions within the cellular model. For the inflammation assay, LPS from *Escherichia coli* O111:B4 (Sigma) was used to elicit a neuroinflammatory response in the microfluidic co‐culture system comprising a BBB and hybrid neurospheroids with dopaminergic differentiation. Treatment was maintained for 48 h, during which the system was monitored for changes in barrier integrity, cellular morphology, and inflammatory signaling. This experimental setup enables systematic evaluation of microglial activation, BBB dysfunction, and neuroinflammatory interactions under controlled, physiologically relevant conditions.

**FIGURE 5:**
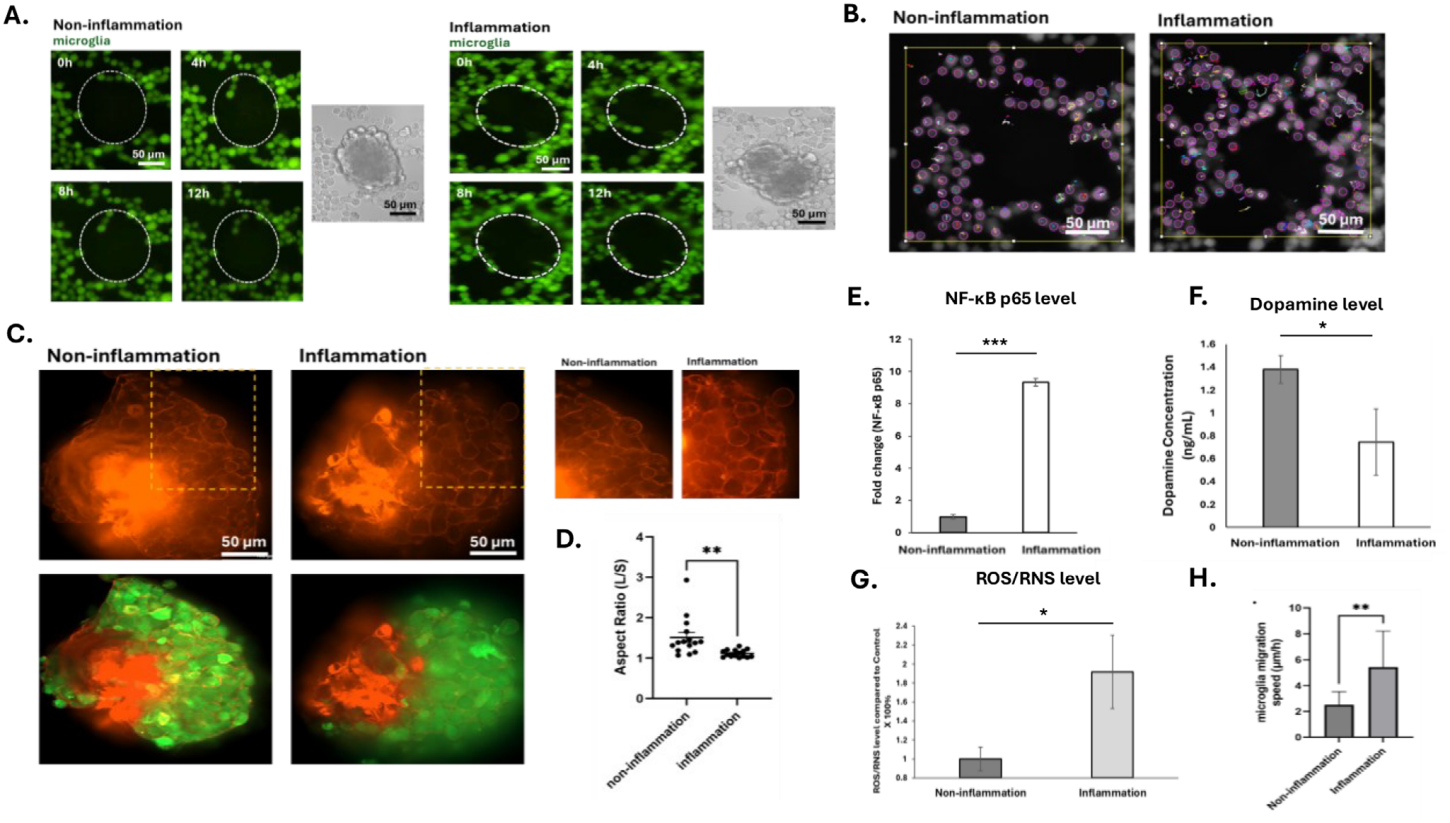
Neuroinflammation‐induced microglial dynamics, neuronal morphological changes, and functional readouts in the BBB‐neurospheroids model. (A) Time‐lapse imaging of microglia (green) in hybrid neurospheroids under non‐inflammatory and inflammatory conditions (0–12 h). Microglia exhibit increased motility and directed movement toward the spheroid core during inflammation. (B) Representative images of microglia moving toward with neurospheroids. (C) Neuronal morphological changes visualized under inflammatory stimulation. (D) Quantification of microglia aspect ratio demonstrating significant shape deformation in the inflammation group. (E) NF‐kB p65 showing elevated level in stimulated spheroids compared to controls. (F) Dopamine measurements illustrating reduced dopaminergic output in inflammation. (G) ROS generation in neurospheroids, demonstrating elevated oxidative stress during inflammatory activation. (H) Quantification of microglial infiltration confirming increased penetration into neuronal regions during inflammatory activation. The results are presented as follows: ^*^ *p* <0.05, ^**^ *p* <0.01, ^***^ *p* <0.001.

Prior to hybrid neurospheroid formation, microglia were labeled with CellTracker Green CMFDA dye (Thermo Fisher Scientific) to enable live‐cell tracking. Microglial morphology was assessed as a structural correlate with its activation. In this experiment, spheroids were seeded together with a functional BBB in microfluidic devices (Figure 5A,B). Mean migration speed was calculated for each condition. Microglia in the non‐inflammatory state migrated at an average speed of 2.3 µm/h, whereas under inflammatory stimulation their migration speed increased to 5.4 µm/h. Representative time‐lapse images demonstrate enhanced motility in the inflammatory group compared with controls (Figure 5C,D,H).

Morphometric analysis in ImageJ quantified the aspect ratio of individual cells from confocal microscopy slices, allowing discrimination between the ramified morphology characteristic of homeostatic microglia and the amoeboid morphology associated with inflammatory activation. Under non‐inflammatory conditions, microglia predominantly exhibited elongated, polygonal morphologies, by cytoskeletal reorganization and process extension, reflecting a shift toward a ramified phenotype, whereas inflammatory stimulation resulted in a marked shift toward a rounded, amoeboid phenotype (Figure 5C).

To further evaluate microglial activation within the microfluidic model, we performed immunofluorescent staining for CD16, a canonical marker of activated microglia (Figure S13). CD16 expression was significantly increased under inflammatory conditions compared with controls, confirming robust microglial activation. NF‐κB p65 (RelA), a key transcription factor driving inflammatory signaling, was also assessed. NF‐κB p65 levels increased significantly (∼9‐fold) following LPS stimulation compared with untreated controls, while control devices maintained low baseline expression throughout the 48‐h culture period (Figure 5E). These findings confirm that the platform reliably models inflammatory signaling through robust NF‐κB pathway activation.

Dopaminergic neurons synthesize, store, and release dopamine as their principal neurotransmitter; therefore, intracellular dopamine levels (or dopamine release into the culture media) serve as a direct measure of neuronal activity and metabolic function. Pro‐inflammatory stimuli such as LPS or cytokines (TNF‐α, IL‐1β, IL‐6) activate NF‐κB signaling, leading to oxidative stress, mitochondrial dysfunction, and downregulation of enzymes essential for dopamine synthesis. Consequently, intracellular dopamine content is expected to decrease under inflammatory conditions. Consistent with this expectation, intracellular dopamine levels were quantified, and in the non‐inflammatory control group (48 h untreated), dopamine concentration was ∼1.38 ng/mL, whereas in the LPS‐treated group (1 µg/mL for 48 h), dopamine levels decreased to ∼0.75 ng/mL (Figure 5F). These results indicate that LPS stimulation significantly impairs dopaminergic function within the microfluidic model.

Analysis of ROS/RNS levels revealed a significant increase in the LPS‐treated (48 h) group compared with untreated controls. Relative fluorescence intensity increased by approximately twofold under inflammatory stimulation, consistent with elevated free radical production (Figure 5G,H). These findings demonstrate that neuroinflammatory conditions within the microfluidic culture system induce oxidative stress, as reflected by increased ROS/RNS levels in the culture media.

Collectively, these results demonstrate that microglia undergo structural and functional activation in response to inflammatory stimulation within the microfluidic co‐culture system. Quantitative tracking revealed significantly enhanced microglial migration under inflammatory conditions, while immunofluorescent staining confirmed upregulation of the activation marker CD16. Morphological analyses further revealed cytoskeletal remodeling, including F‐actin reorganization and a marked reduction in aspect ratio, indicative of a transition toward an amoeboid, activated phenotype. Together, these findings validate the platform as a robust and physiologically relevant model for studying microglial activation, migration, and morphological transitions during neuroinflammation.

### 2.3 Functional Validation Using Neuroinflammatory Stimuli

#### 2.3.1 Time‐Resolved Cytokine Profiling and Functional Readouts of Neuroinflammation

**FIGURE 6:**
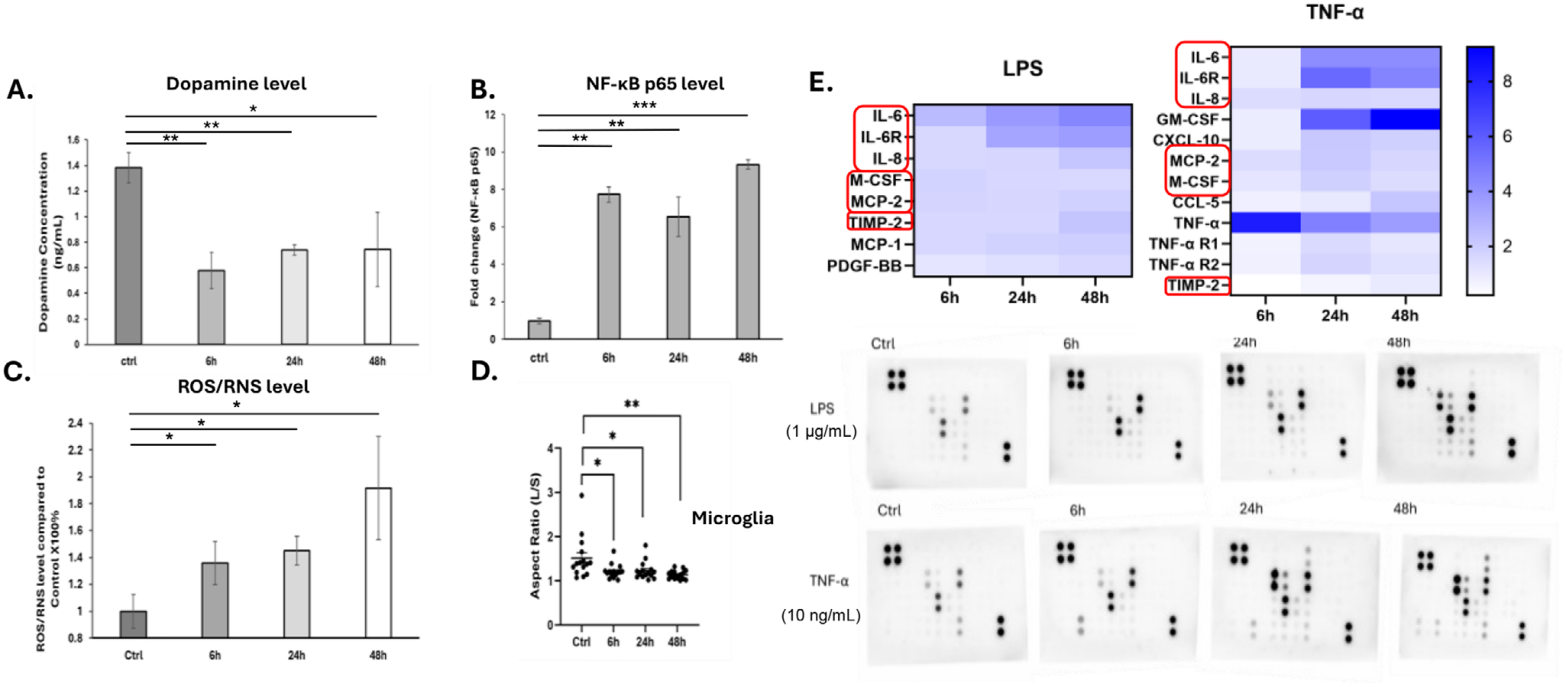
Time‐course analysis of inflammatory responses in the microfluidic neurovascular model. (A)‐(D). Cell lysates or conditioned media were collected from microfluidic device after treatment with LPS for 6, 24, and 48 h, with 48 h untreated devices serving as the non‐inflammatory control. (A) Intracellular dopamine content, (B) NF‐κB p65 levels, (C) ROS/RNS production was evaluated. (D) Microglial morphology was evaluated by Phalloidin staining of F‐actin, and the cell aspect ratio was quantified in ImageJ. (E) Time‐course cytokine array analysis of microfluidic cultures stimulated with LPS (1 µg/mL) or TNF‐α (10 ng/mL). Upper panels show the heatmap of the quantitative analysis of each cytokine's relative expression level. 6 cytokines were detected as elevated for both the LPS and TNF‐α groups (highlighted in red). Lower panels display representative chemiluminescent blot images from control and treated groups. The results are presented as follows: ^*^ *p* <0.05, ^**^ *p* <0.01, ^***^ *p* <0.001.

We performed inflammatory endpoint assays at 6, 24, and 48 h following LPS treatment. Dopamine levels in dopaminergic neurons declined as early as 6 h after stimulation and remained suppressed through 48 h (Figure 6A). A similar temporal pattern was observed for NF‐κB p65 (RelA) activation, which increased significantly by 6 h (∼9‐fold relative to control) and remained elevated over time (Figure 6B). ROS/RNS production was also increased from 6 h onward, with a progressive rise through 48 h (Figure 6C). Consistent with these molecular changes, microglial morphology began to shift from a ramified to an amoeboid phenotype by 6 h, indicating rapid activation and migration (Figure 6D). These multiparametric analyses across time points demonstrate a coordinated inflammatory response characterized by NF‐κB activation, oxidative stress, impaired dopaminergic function, and microglial activation.

To further characterize inflammatory signaling in the microfluidic model, we performed time‐course cytokine profiling (Figure 6E). Devices were stimulated with LPS (1 µg/mL) or an alternative inflammatory stimulus, TNF‐α (10 ng/mL), and conditioned media were collected at 6, 24, and 48 h for cytokine array analysis (Figure S14). TNF‐α was included to assess whether the system responds to a distinct and more potent pro‐inflammatory cue beyond bacterial endotoxin, thereby validating model sensitivity and versatility. TNF‐α elicited a stronger overall cytokine response than LPS, but induced a different set of inflammatory mediators. Semi‐quantitative analysis indicated that TNF‐α stimulation markedly upregulated 12 cytokines and growth factors, with GM‐CSF showing the greatest increase (∼9‐fold). Other molecules—including IL‐6, IL‐6R, IL‐8, MCP‐2, M‐CSF, and TIMP‐2—were also elevated. Most mediators peaked at 24 h and declined thereafter. Notably, TNF‐α was highest at 6 h and decreased over time, whereas other chemokines (e.g., CCL‐5, CXCL‐10) remained comparatively low (1.3–1.4‐fold changes).

In comparison, LPS stimulation elicited a weaker and more delayed cytokine response. Only eight cytokines exhibited modest increases, with expression levels remaining substantially lower than those induced by TNF‐α. Six molecules were commonly upregulated in both the TNF‐α– and LPS‐treated groups (Figure 6E; Figure S15A,B). These mediators are canonical downstream targets of NF‐κB and MAPK signaling pathways and are associated with endothelial and glial activation, leukocyte recruitment, and extracellular matrix remodeling [78]. Notably, TNF‐α was not detected under LPS stimulation. This absence is likely attributable to the characteristics of the HMC‐3 microglial cell line used in this system and is consistent with prior reports under comparable LPS treatment conditions [79]. Overall, these results indicate that TNF‐α induces a more robust and broader pro‐inflammatory response than LPS in this model, reflecting differential activation pathways and distinct sensitivities of the co‐culture system to cytokine vs. endotoxin exposure. Overall, these findings demonstrate that the microfluidic platform can discriminate between endotoxin‐driven and cytokine‐driven inflammatory programs, with each stimulus producing a distinct cytokine release profile.

### 2.4 Integration of TEER Measurements for BBB Barrier Monitoring

#### 2.4.1 Inflammation‐Dependent Disruption and Recovery of BBB Integrity Revealed by TEER Dynamics

TEER measurements were performed in two experimental batches using fully developed 3D BBB models. Each batch comprised six devices generated over a period exceeding one month. Models were divided into three groups: LPS, TNF‐α treatment, and no treatment as a control. Inflammatory groups were treated with either LPS (1 µg/mL) or TNF‐α (10 ng/mL). TEER measurements were collected at 0, 6, 24, and 48 h, in triplicate, with a 5‐min resting period between measurements (Figure S16). These experiments revealed three key findings. First, both LPS‐ and TNF‐α‐induced inflammation resulted in an initial decrease in TEER followed by a recovery phase. LPS treatment caused a more prolonged disruption of barrier integrity, with a 10%–30% greater reduction in resistance compared with TNF‐α (Figure S16A). Second, TNF‐α treatment induced a steeper initial drop in resistance, followed by complete recovery or resistance values exceeding baseline, indicating restoration of barrier integrity (Figure S16B). Third, control devices exhibited a continuous increase in resistance during the first 12 h, after which TEER values stabilized (Figure S16C). We note that the measured TEER amplitude varied across devices due to differences in microvascular network density; however, all devices demonstrated time‐dependent changes in resistance, indicating sensitivity to LPS and TNF‐α exposure.

**FIGURE 7:**
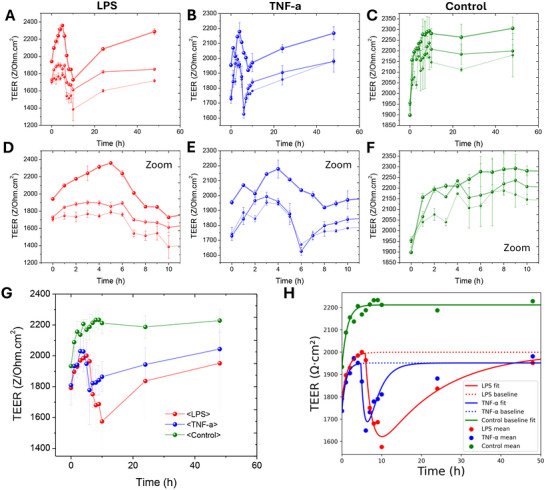
Time‐course analysis of inflammatory responses in the microfluidic neurovascular model measured using TEER sensors. (A) Time‐dependent TEER measurements of three 3D BBB devices following exposure to LPS (1 µg/mL) (*n* = 3). (B) Time‐dependent TEER measurements of three 3D BBB devices following exposure to TNF‐α (10 ng/mL) (*n* = 3). (C) Time‐dependent TEER measurements of three 3D BBB devices used as controls, without exposure to inflammatory agents, measured under the same conditions as the treated samples. (*n* = 3). (D) Magnified view of TEER measurements from LPS‐treated samples, showing an initial increase in resistance followed by a gradual decrease, reaching a minimum at approximately 10 h. (E) Magnified view of TEER measurements from TNF‐α–treated samples, showing a faster decrease in resistance, reaching a minimum at approximately 6 h. (F) Additional magnified view of TEER measurements of three 3D BBB devices used as controls, without exposure to inflammatory agents. (G) Averaged TEER measurements across all samples within each experimental group. Standard deviation (SD) was calculated and plotted on the graph, revealing minor overlaps between the LPS‐, TNF‐α‐, and control‐treated groups. (H) Data are presented as mean ± SEM (*n* = 3 independent chips). LPS and TNF‐α differentially modulate barrier integrity, producing distinct temporal patterns of disruption and recovery in TEER measurements. Statistical analysis is described in Figure S17.

To further investigate the dynamics underlying these responses, a second batch of 3D BBB models was generated and treated identically. Devices were again divided into LPS, TNF‐α, and control groups and exposed to LPS (1 µg/mL) or TNF‐α (10 ng/mL) (Figure 7). In this batch, TEER measurements were acquired at higher temporal resolution—0, 1, 2, 3, 4, 5, 6, 7, 8, 9, 10, 24, and 48 h—in triplicate. High‐resolution TEER profiling revealed modest inter‐device variability, which could associate with micro vessels density and packaging, while confirming the trends observed in the initial experiments. LPS‐treated devices exhibited an initial increase in resistance, followed by a decline beginning at approximately 6 h and reaching a minimum at ∼10 h (Figure 7A,D). TNF‐α‐treated devices displayed a similar pattern; however, the decrease in resistance occurred earlier, at approximately 3 h—approximately twice as fast as observed for LPS‐treated devices (Figure 7B,E). In contrast to LPS, resistance recovery in the TNF‐α group began as early as 6 h, indicating a more transient disruption of barrier integrity. Control devices reached a resistance plateau after approximately 4 h of measurement (Figure 7C,F). Collectively, these high‐resolution TEER measurements highlight the dynamic behavior of the 3D BBB model, characterized by an initial barrier disruption followed by either complete recovery (TNF‐α) or partial recovery (LPS).

Finally, TEER data from the second batch were averaged and fitted using the proposed analytical models (Figure 7G,H). The averaged profiles confirmed that the magnitude of resistance loss, the depth of the minimum, and the recovery kinetics were highly specific to each inflammatory condition. TEER changed significantly over time (*p* < 0.001) and showed a significant Treatment × Time interaction (*p* < 0.001), indicating distinct temporal barrier‐disruption dynamics for LPS vs. TNF‐α (see Methods). To model the TEER response, the signal was decomposed into two components: (i) a gradual increase in resistance associated with maturation of the 3D BBB tissue, and (ii) a transient resistance “valley” corresponding to inflammation‐induced barrier disruption and subsequent recovery. These differences are commonly quantified using the FWHM, which differed between conditions (FWHM = 15.8 h for LPS and 4.29 h for TNF‐α), which is ∼3.6 times the inflammation stress. Our results support the rapidly developing field of microphysiological systems [80, 81, 82]. In addition, our future work will address the relationship between vascular network density and TEER amplitude responses, which could further improve the application of TEER sensors for understanding the properties of different inflammation models or drugs.

#### 2.4.2 Quantitative Comparison of LPS‐ and TNF‐a–Induced Barrier Disruption Kinetics Using Analytical TEER Modeling

To enable direct quantitative comparison of inflammatory barrier disruption dynamics, we fit the averaged TEER time courses for control, LPS, and TNF‐α conditions using an analytical model that decomposes TEER into (i) a baseline maturation term and (ii) a delayed transient disruption (“dip”) term capturing inflammatory injury and recovery. The TEER trajectory *R*(*t*)was modeled as: *R*(*t*)  = *R*_base_ (*t*) − Dip(*t*). The same time the baseline maturation term (Control drift): $R_{\mathrm{base}}\left(t\right)=R_{0}+G\left(1e^{-k_{g}t}\right)$, where *R*_0_is TEER at *t*  =  0, *G*(Ω·cm^2^) is the asymptotic baseline gain, and *k_g_* (h^−1^) is the maturation rate constant. The detailed function for the inflammatory disruption term (delayed transient dip):

$$\mathrm{Dip}\left(t\right)=A\left(1e^{-k_{1}\left(t-t_{\mathrm{lag}}\right)}\right)e^{-k_{2}\left(t-t_{\mathrm{lag}}\right)}\mathrm{for}\;t\geq t_{\mathrm{lag}},$$

and Dip(*t*)  =  0 for *t* < *t*_lag_. Here, *A*(Ω·cm^2^) defines the disruption amplitude scale, *k*_1_(h^−1^) is the disruption onset rate, *k*_2_(h^−1^) is the recovery/relaxation rate, and *t*_lag_(h) is the delay between stimulation and measurable TEER disruption. Therefore, the control curves were fit using *R*(*t*)  = *R*_base_ (*t*)only (i.e., *A*  =  0). Controls were fit using the baseline term alone. Fitting the averaged control curve yielded a monotonic stabilization profile consistent with baseline maturation (G = 277 Ω·cm^2^, k_g_∼0.73), confirming progressive barrier tightening in the absence of inflammatory stimulus. In contrast, both LPS and TNF‐α exhibited non‐monotonic trajectories characterized by early maturation followed by disruption and recovery. For the averaged LPS response, the fitted parameters indicated a pronounced disruption with dip amplitude A = 561 (Ω·cm^2^) and onset delay *t_lag_* ∼5.9 h, followed by disruption *k_1_* ∼10.0 (h^−1^), and recovery with *k_2_* ∼0.07 h^−1^. This behavior corresponded to an approximate 10% decrease relative to the baseline *R*_0_, with the model‐predicted minimum occurring near 10 h. These results are consistent with a robust but partially reversible barrier impairment induced by LPS.

For the averaged TNF‐α response, the disruption component was comparatively smaller and less well constrained when all dip parameters were allowed to vary freely, reflecting the relatively shallow perturbation present in the averaged TEER trace. The TNF‐α response exhibited a smaller disruption magnitude than LPS with A = 345 Ω·cm^2^ and an earlier onset delay (*t_lag_* = 3.8 h), followed by disruption *k_1_* ∼0.5 (h^−1^), and slower recovery (*k_2_* = 0.38 h^−1^). Together, these results indicate that, when onset kinetics are normalized, LPS drives a stronger disruption amplitude, whereas TNF‐α produces a more persistent perturbation with slower relaxation.

The above findings suggest that TNF‐α activates a stronger but self‐limited inflammatory response, whereas LPS induces a slower, longer‐lasting disruption, reflecting different cellular sensing mechanisms and kinetics of barrier remodeling in response to cytokine vs. endotoxin exposure. Combining the cytokine data and TEER data, the findings suggest that TNF‐α primarily drives acute, high‐amplitude inflammatory signaling with reversible barrier effects, whereas LPS engages slower, immune‐mediated amplification pathways that produce persistent functional consequences despite lower cytokine output. To further investigate the relationship between cytokine expression in relation to TEER dynamics, we performed a Pearson correlation analysis using GraphPad Prism. Data collected at 6, 24, and 48 h following TNF‐α and LPS stimulation were analyzed separately to identify factors most strongly associated with BBB disruption. After correcting for the direction of TEER change (decrease representing barrier loss), Pearson analysis revealed expected negative correlations between cytokine levels (IL‐8, MCP‐2) and TEER (r (IL‐8) = −0.7097 (LPS)/‐0.5072 (TNF‐α) to r (MCP‐2) = −0.9454 (LPS)/‐0.6812 (TNF‐α), *p* <0.05), confirming that increased inflammatory signaling corresponds to reduced barrier integrity, as described earlier for IL‐8 increased BBB permeability [83]. MCP‐2 likely contributes to BBB dysfunction via an indirect mechanism, whereby it recruits peripheral immune cells that subsequently secrete cytokines, chemokines, and proteases that compromise endothelial tight‐junction integrity. The observed associations between cytokine levels and TEER decline indicated potential roles of IL‐8 and MCP‐2 in barrier modulation [84, 85]. Further investigation into whether these chemokines directly or indirectly contribute to BBB dysfunction is necessary. Moreover, future experiments will assess barrier function using 40 kDa dextran permeability at the lowest TEER time point post‐inflammation to further validate endothelial disruption.

In conclusion, TEER measurements showed stimulus‐dependent changes in neuroinflammation. TNF‐α caused a rapid but transient decrease, whereas LPS produced a delayed and more sustained reduction. Cytokine analysis indicated increased secretion of IL‐8, and MCP‐2 that coincided with periods of lower TEER, suggesting an association between inflammatory signaling and barrier integrity. A coordinated inflammatory response also characterized by NF‐κB activation, oxidative stress, impaired dopaminergic function, and microglial activation.

This model captures the principal features of BBB resistance dynamics under inflammatory challenge, systematic deviations between experimental TEER measurements and fitted trajectories reveal biologically meaningful differences in how TNF‐α and LPS perturb barrier integrity. In TNF‐α–treated BBBs, the data show rapid junctional disruption followed by delayed and sometimes baseline recovery, which is underestimated by the fitted recovery term. This behavior likely reflects secondary, transcription‐dependent processes—such as actomyosin remodeling, tight‐junction protein reorganization, and adaptive endothelial–astrocyte signaling—that evolve on longer timescales than acute cytokine receptor activation and are not explicitly represented in simplified kinetic models [3, 86, 87]. By contrast, LPS exposure produces a deeper and more persistent TEER reduction experimentally than predicted by the model, consistent with sustained Toll‐like receptor 4 (TLR4)–NF‐κB signaling, oxidative stress, and prolonged cytokine cascades that impair junctional reassembly and limit functional recovery [88, 89, 90]. Importantly, the divergence between experimental data and fitted curves highlights the novelty of our framework, which explicitly separates barrier disruption and repair as competing dynamic processes rather than treating TEER changes as a single decay or recovery event. While the extent of barrier disruption correlates with inflammatory cytokine levels, the duration of barrier dysfunction does not necessarily follow the same pattern, reflecting differences between direct and immune‐amplifying mechanisms. This kinetic decomposition enables discrimination between cytokine‐ and endotoxin‐driven neuroinflammatory phenotypes based on temporal signatures, not solely magnitude, and provides a foundation for future extensions incorporating delayed inflammatory terms, adaptive recovery rates, and integration with orthogonal biophysical readouts [48, 91, 92].

## 3 Conclusions

We established a perfusable and TEER‐compatible 3D‐HIC‐BBB MPS model that achieves relevant permeability, integrated it with dopaminergic neurospheroids and microglia, and demonstrated robust neuroinflammatory responses to LPS and TNF‐α. The platform captures multiple hallmarks of inflammation—including NF‐κB activation, oxidative stress, microglial migration and morphological change, altered neuronal dopamine activity, and cytokine secretion—while maintaining cellular viability and functional readouts. By combining vascular, neuronal, and immune elements within a single microfluidic system, this work provides a versatile tool for investigating neurovascular dysfunction. Importantly, these advances lay the groundwork for future research in neurological diseases, enabling systematic study of how pathological insults and inflammatory signaling converge to disrupt BBB integrity and neuronal function.

## 4 Experimental Section

### 4.1 Device Design and Fabrication

The device consists of two parallel gel channels and two media channels, separated by pillars as reported earlier [93]. The master mold was designed in AutoCAD 2016 (Autodesk, Mill Valley, CA) and made via photolithography on a silicon wafer using photoresist, SU‐8 100 (Kayaku Advanced Materials, Westborough, MA), to generate patterns of microstructure. PDMS devices were fabricated by filling the mold with a mixture of elastomer base and curing agent of SYLGARD 184 Silicone Elastomer in a ratio of 10:1 (w/w) (base to curing agent), prior to being degassed and cured under a vacuum oven (VWR, Radnor, Pennsylvania). Once cured, PDMS devices were cut from the mold. Inlets to the devices were punched using biopsy punches with 4 mm diameter holes for the media ports, and 1.5 mm diameter holes for the gel ports, and the central region of the BBB gel side was punched for two holes using a 2 mm diameter biopsy, to facilitate the integration of TEER sensor electrodes (with an open top). After that, the devices were bonded to glass coverslips using air plasma for 2 min, and the channel dimension was shown in Figure S1.

### 4.2 Cell Culture and Maintenance

For BBB, human primary brain endothelial cells (HBMEC, Angio‐Proteomie, cat. no. cAP‐0002), human astrocytes (AC) from cerebral cortex (ScienCell, cat. no. 1800), and human brain vascular pericyte (PC) (ScienCell, cat. no. 1200) were used. All the primary cells were used between passages 5 and 7. HBMECs were cultured in vascular endothelial growth factor (VEGF)‐supplemented endothelial growth medium (Lifeline Cell Technology, Frederick, MD, USA), with the flasks coated with collagen type I. AC were cultured in Astrocyte Medium (ScienCell, cat. no. 1801), and PC in Pericyte Medium (ScienCell, cat. no. 1201). Human neural progenitor cells (NPCs) were obtained from ATCC (ACS‐5004). They were expanded in 2D culture until ∼90% confluence in DMEM/F‐12 medium (ATCC cat. no. 30–2006) supplemented with Neural Progenitor Cell Expansion Kit components (ATCC cat. no. ACS‐3003), coated with Matrigel. HMC‐3 human microglial cells (ATCC, CRL‐3304) were maintained in EMEM (ATCC‐30‐2003) plus 10% fetal bovine serum (FBS). Cells were maintained at 37°C with 5% CO_2_ in a standard incubator. 0.05% of trypsin‐EDTA was used for AC, PC, and HMC‐3 detachment, TrypLE express enzyme (Thermo Fisher Scientific) was used for EC detachment, and Accutase was used for NPCs detachment. The media was refreshed every other day.

### 4.3 3D Hybrid Spheroids Generation

Three‐dimensional dopaminergic neuron spheroids were generated from NPCs following the manufacturer‐recommended protocol (ATCC). NPCs were dissociated using Accutase and seeded into ultra‐low attachment (ULA) round‐bottom 96‐well plates (Corning Costar) at a defined cell density (2000 cells/well). Within 12–24 h of plating, NPCs self‐aggregated to form uniform spheroids. NPC expansion media was gently changed every two days by allowing spheroids to settle and carefully aspirating half of the medium without disturbing the aggregates. After 7–10 days of spheroid formation, cultures were transferred to dopaminergic differentiation medium (ATCC NPC Dopaminergic Differentiation Kit, cat. no. ACS‐3004) and maintained for an additional 21 days following the manufacturer's differentiation protocol. To form hybrid neurospheroids with microglia, on differentiation day 19, microglia were added to dopaminergic neuronal spheroids at a 1:5 ratio (∼400 microglia per well) and allowed to self‐assemble in suspension culture for 48 h. The hybridization period media consisted of a 1:1 mixture of microglia culture media and dopaminergic differentiation media.

### 4.4 Device Seeding

Thrombin stock solution of 100 U/mL was prepared in 0.1% w/v bovine serum albumin (BSA) solution (Sigma–Aldrich, St. Louis, MO), of which 40 µL was added to 960 µL of EC growth media to make a final concentration of 4 U/mL, and kept on ice. Fibrinogen from bovine solution (Sigma Aldrich, St. Louis, MO) was made to a concentration of 6 mg/mL in Dulbecco's phosphate‐buffered saline (DPBS). ECs, PC and AC were re‐suspended in thrombin solution separately at a density of 3.0 × 10^7^ cells/mL, 3 × 10^6^ cells/mL, and 6 × 10^6^ cells/mL, respectively (at a ratio of 10: 1: 2). Cell‐suspended thrombin solution was mixed in equal volumes for each cell type and then mixed with equal volumes of fibrinogen solution. The BBB vascular structure subsequently underwent a self‐assembly process for an additional 4 days, and the vasculature was supplied with endothelial growth media containing VEGF‐165 (50 ng/mL, Peprotech). On day 4 (differentiation day 18), a monolayer of ECs (1 × 10^6^ cells/mL) at the medium–gel channel interface (vascular opening side) was introduced to restrain direct diffusion from the cell culture medium into the fibrin gel matrix (to ensure all substances move into the luminal side of vasculature without leaking into the basal side of the vessels–gel region). Formation of the BBB with only one side of the opening was demonstrated in the Supporting Information. On day 5, the culture medium was switched back to standard EC growth medium, and the BBB structure reached maturity by day 7.

To establish a co‐culture model of the BBB and hybrid neurospheroids enriched with dopaminergic neurons, Neurospheroids were cultured using the methods shown above. And 7 days before harvesting (day 21). BBB was first formed in microfluidic devices with fully developed BBB networks by day 21. On day 28, the hybrid neurospheroids were introduced into the central compartment of the microfluidic BBB chip to form a 3D co‐culture system. By Day 30, the platform is ready for evaluation of neurovascular interactions.

### 4.5 Immunofluorescent Staining

The BBB cell types and the auditory hair cell type were stained with CD31 (EC, ab32457), GFAP (AC, ab4674), PDGFR‐β (PC, ab69506), ZO‐1 (tight junction, ab221547). For neurospheroids, tyrosine hydroxylase (TH, ab137869), TUJ1 (βIII‐tubulin, ab78078), PAX6 (ab195045), and Nestin (ab22035), IBA1 (ab283319) and CD11b (ab52478), and CD16 (ab246222) was for microglia characterization. All the primary antibodies were purchased from Abcam unless otherwise stated (Waltham, MA). Briefly, cell culture media in the device was removed, and cells were rinsed by 1x DPBS and fixed by 4% paraformaldehyde (PFA) (Fisher Scientific, Waltham, MA) for 15 min at room temperature. 0.1% Triton X‐100 (Sigma–Aldrich, St. Louis, MO) was then applied for another 15 min prior to adding cell blocking solution (5% w/v BSA dissolved in 1x DPBS) for 1 h at room temperature. Then the cells were stained with primary antibodies overnight at 4°C. On the next day, secondary antibodies (1:200) Alexa Fluor 647 (goat anti‐mouse IgG), Alexa Fluor 488 (goat anti‐chicken IgG), and Alexa Fluor 594 goat anti‐rabbit IgG), Invitrogen, Carlsbad, CA), were applied for 1 h at room temperature for the respective samples, followed by subsequent DPBS washing. The spheroids were then fixed and stained with GFP‐Phalloidin (Thermo Fisher Scientific) for F‐actin. Confocal z‐stack images were made using a 5 µm step size and about 20–25 xy‐slices acquired per image. Fluorescent images were obtained by fluorescent microscopy (Olympus (IX83), Tokyo, Japan) and Cytation10 confocal imager (Agilent, Santa Clara, CA).

### 4.6 Harvest Spheroids/Cellular Contents from Microfluidic Devices

To recover 3D neural constructs from microfluidic devices, chips containing BBB/dopaminergic neuron–microglia co‐cultures were placed in a biosafety cabinet and the hydrogel region was carefully exposed by cutting along the gel boundary using a sterile scalpel, avoiding disruption of the matrix. A Liberase digestion solution (0.5 mg/mL in pre‐warmed DMEM) was added to fully cover the gel region (∼100 µL/device), and the cut segment was repositioned to ensure complete exposure of the hydrogel to the enzyme. Devices were incubated at 37°C for 30 min with gentle tapping every 5–10 min to facilitate hydrogel breakdown, with optional extension in 5–10 min intervals if fragments remained (total digestion time <60 min). Following enzymatic release, the liberated cell pellets were collected into pre‐labeled low‐binding microcentrifuge tubes on ice, and devices were rinsed twice with cold PBS (200 µL each) to maximize recovery. Neurospheroids were isolated using 100 µm strainers and gently back‐flushed with ∼5 mL PBS to retain spheroid integrity, then centrifuged at 150 × g for 5 min at 4°C. To harvest the entire cellular contents for downstream analysis, no cell strainers were used before centrifugation, and the cell pellets were collected for cell lysis.

### 4.7 Ca^2+^ Assay

Calcium signaling was assessed to evaluate neuronal functionality using a fluorescence‐based Ca^2^^+^ indicator (Fluo‐4 Direct Calcium Assay Kit, Molecular Probes, cat. no. F10471). Spheroids (∼10 000 cells each) were transferred to individual wells of a 96‐well black‐walled plate, with five spheroids per well and approximately five replicate wells per condition. Following dye loading according to the manufacturer's instructions, fluorescence measurements were collected every 20 s for 10 min (30‐time points total) using a microplate fluorescence reader set to 485 nm excitation and 520 nm emission. Calcium activity for each well was quantified as ΔRFU, defined as the difference between the maximum and minimum fluorescence values recorded during the imaging period.

### 4.8 Enzyme‐Linked Immunosorbent Assay (ELISA)

Dopamine level was quantified by ELISA from engineered BBB–dopaminergic neuron–microglia constructs following LPS stimulation. Devices were cut under sterile conditions, and tissues were harvested using the method mentioned above. Pellets from three devices were pooled per replicate and were lysed on ice in the kit‐supplied dilution buffer (ab285238, Abcam), briefly sonicated, and cleared by centrifugation (12 000–16 000 × g, 10 min, 4°C). The experiments were performed following the manufacturer's protocol. Absorbance was measured at 450 nm with a 600 nm reference using a microplate reader, and cytokine concentrations were calculated from standard curves using the kit‐specified analysis method. For NF‐κB p65 ELISA, cell pellets were collected using the same method, but a lysis buffer was used. Basically, the cell pellets were incubated with ice‐cold kit lysis buffer (150 µL/pellet; protease ± phosphatase inhibitors added as permitted), vortexed for 3–5 s, and incubated on ice for 10–15 min, followed by clarification at 12 000–16 000 × g for 10 min at 4°C; supernatants were collected into low‐bind tubes, and total protein concentration was measured by BCA assay for normalization. Experiments were performed using an ELISA kit (ab176648) according to the manufacturer's protocol. Cytokine concentrations were calculated from standard curves generated by linear fit.

### 4.9 ROS Assay

Reactive oxygen and nitrogen species (ROS/RNS) levels were quantified from conditioned media collected from BBB–dopaminergic neuron–microglia microfluidic devices. One device constituted one biological replicate, with three replicates per group. Cell‐culture supernatants were collected from both sides of the device into pre‐chilled low‐bind tubes, followed by centrifugation (10 000 × g, 5 min, 4°C), and either assayed immediately or snap‐frozen at −80°C. ROS/RNS concentrations were measured using a fluorescence‐based assay (ab238535, Abcam) according to the manufacturer's instructions. Briefly, samples were diluted in assay buffer as recommended, loaded in duplicate alongside DCF standards, incubated with the kit catalyst and DCF reagent, and fluorescence was recorded at 480 nm excitation and 530 nm emission using a microplate reader.

### 4.10 Cytokine Array

Secreted inflammatory factors were profiled from the BBB–dopaminergic neuron–microglia microfluidic system using a cytokine array. Each device contained ∼400 µL of medium (200 µL per channel), and media from three devices were pooled to form one biological replicate (1 mL total); two biological replicates were collected per condition (a total six devices/condition). Media were collected and transferred into pre‐chilled low‐bind tubes, clarified by centrifugation (10 000 × g, 5 min, 4°C), and either used immediately or snap‐frozen at −80°C. Cytokine profiling was performed using a membrane‐based human cytokine array kit (AAH‐CYT‐3) according to the manufacturer's instructions (Cytokine identities can be found in Figure S14). Briefly, membranes were blocked, incubated with pooled media samples, washed, and sequentially incubated with biotinylated detection antibodies and HRP‐streptavidin, followed by chemiluminescent substrate development. Membranes were imaged using a CCD imager, and signal intensities were quantified by densitometry (ImageJ or kit‐provided software), normalized to array‐printed positive controls, and compared across control and treated conditions. Briefly, chemiluminescent images were imported, converted to 8‑ or 16‑bit, and uniformly contrast‑adjusted without clipping. A set of same‑sized circular ROIs was applied to all cytokine spots and to the on‑membrane positive controls; identical background ROIs were placed in blank areas adjacent to each spot. For each spot, the integrated density (sum of gray values within the ROI) was recorded and background‑subtracted. To enable comparison across membranes, background‑corrected values were normalized to the average positive‑control signal on the same membrane, yielding a dimensionless, semiquantitative intensity. Duplicate spots per analyte were averaged, and results were reported as relative fold change vs. the non‑inflammatory control

### 4.11 Vessel Permeability Analysis

Media was completely removed and the devices were perfused with 10 kDa Texas Red dextran (ThermoFisher, Waltham, MA) by introducing 40 µL of dextran into one channel. A transient temporary drop in pressure was applied across the gel region and was maintained for approximately 30 s. Once the vasculature was fully perfused, an equal volume of 1 × DPBS was applied into the opposing media channel to stop the flow. After stabilization (∼2–3 min), 3D time‐lapse confocal images were obtained (2 × 6 min intervals). The analysis was conducted using ImageJ. Maximum projection images (of the dextran channel at *t* = 0) were used to generate a separation between the vessel perimeter (*Pv*) and extravascular tissue area (*A_T_*). The effective permeability *P* (cm/s) of the microvasculature to 70 kDa dextran could be calculated using:

$$P\;\left(t\right)=\frac{A_{T}\;\left(I_{T_{f}}-I_{T_{o}}\right)}{P_{v}\;t\;\left(I_{V_{o}}-I_{T_{o}}\right)}$$

where *I* is fluorescence intensity, with *I_T0_* and *I_Tf_* being the initial and final fluorescence intensities in the gel, respectively, and *I_V0_* being the initial intensity in the vessels.

### 4.12 Time‐Lapse Imaging/Analysis

Microglia were labeled with CellTracker Green CMFDA Dye (Thermo Fisher Scientific) to allow live‐cell tracking. After adding microglia to neurospheroids, microglia migration was recorded by time‐lapse imaging at 20‐min intervals for up to 12 h. Trajectories of individual microglia were extracted in ImageJ (TrackMate plugin), and the mean migration speed (µm/h) was calculated from the software.

### 4.13 Microglial Morphometric Analysis

Live‐cell confocal images of microglia at the spheroid periphery were acquired at specified time points. Individual cells were segmented and traced using ImageJ/Fiji, and morphometric parameters—including soma area, process length, branching, and circularity,—were quantified. Measurements were averaged over *n* = 3 independent experiments, and statistical analysis was performed as described.

### 4.14 Statistical Analysis

For biochemical assays, each sample had four replicates unless otherwise noted. The statistical analyses of sample means were performed using GraphPad Prism software. One‐way ANOVA, complemented by Tukey's pairwise comparisons, was used to evaluate group differences. A *p*‐value less than 0.05 was considered statistically significant.

### 4.15 TEER and Surface Area

GFP expression VE‐cadherin HBMECs were obtained from Angioproteomie (cAP‐0002VE‐CAD‐GFP). Areal endothelial coverage was quantified from this cell type to normalize TEER. The cells were imaged by Cytation10 confocal imager (Agilent, Santa Clara, CA). For each device, z‐stacks of the endothelial surface were acquired (≤ 5 µm step), and a maximum‐intensity projection was generated to produce a projection image of the fluorescent junctional network representing the vessel surface. The vessel area (%) was computed as 100 × [VE‐cadherin–positive pixels/total region‐of‐interest (ROI) pixels] (ROI area (cm^2^)). TEER was measured immediately before imaging.

The normalized surface area of fluorescence‐labeled vessels was calculated as a percentage using ImageJ. For each image (confocal z‐stack image, maximum intensity projection), a 300 µm × 300 µm region was cropped and thresholded to isolate the fluorescent signal. The % vessel area was determined by calculating the ratio of the GFP‐positive area to the total cropped area. Using the known conversion between pixel area and actual area in cm^2^, vessel coverage was normalized and reported as a percentage of the total channel surface. To ensure consistency, all devices were seeded with the same cell density and maintained with identical channel geometry, and images were acquired from 5–7 different regions per device. For each device, the normalized % vessel area is about ∼ 24.3% (For each batch of experiments, we calculated the exact vessel surface area to ensure that functional readouts). Since all devices used the same seeding density and geometry, no significant differences in vessel area were expected across devices, unless influenced by external stimuli such as apoptotic or inflammatory signals.

### 4.16 TEER Measurements

Next, we conducted experiments to measure TEER across microfluidic chips. Once the cells were ready, the samples were removed from the incubator, and placed on a pre‐cleaned working surface and electrodes, disinfected with 70% ethanol (EtOH). The samples were kept at room temperature for at least 15 min. Next, an additional 100 µL of cell culture media was applied to the top side of the chips, which was then covered with a second set of Au electrodes, facing downward toward the chip. After visually confirming that there were no bubbles in the channel, electrical measurements were carried out according to the established procedure, any present bubbles were carefully removed using a pipette. The measurements were performed using a potentiostat, as described previously [94]. All electrical impedance spectroscopy (EIS) measurements were performed using the same setup and top electrodes. The obtained data were normalized to the surface area of each device (width: 2 mm; length: 7 mm), considering 24.3% coverage by the vascular structures calculated from above. We fabricated two samples with identical properties using chips cultured for seven days, following the same strategy described above. Our measurements show good reproducibility, with very similar TEER resistance values between the samples, ranging from 1300 to 1600 Ω·cm^2^. These values are well‐aligned with those reported in the literature for microfluidic BBB systems and are notably higher than the TEER values obtained using the Transwell setup [94, 95], and also in vivo rat brain capillary (∼1462 Ω·cm^2^) [70]. TEER time courses were analyzed using a linear mixed‐effects model to account for repeated measurements within each replicate (random intercept for replicate; fixed effects: Treatment, Time, and Treatment × Time), implemented in R (lme4 package). TEER changed significantly over time (*p* < 0.001) and exhibited a significant Treatment × Time interaction (*p* < 0.001), demonstrating distinct temporal dynamics of barrier disruption for LPS vs. TNF‐α.

## Author Contributions

P.G. and J.B. contributed equally to this work. J.B., O.A.B., and D.S. designed and conceptualized the study. J.B. developed the tissue models and performed assay analyses. P.G. designed the experimental setup for TEER measurements; conducted the TEER measurements; performed the modeling; and wrote the manuscript. R.M.G. and A.M.S. contributed to the development of biological assays and discussed experiments. J.H.L. and P.W.F. prepared the figures. L.C.N. contributed to concept development; participated in meetings; discussed the results; and contributed to data interpretation and analysis. J.F.S. supervised the project, organized NIH meetings, reviewed progress and reporting, and initiated the publication. All authors reviewed and edited the paper prior to submission.

## Funding

National Institutes of Health: 75N950‐24‐C‐00036.

## Conflicts of Interest

The authors declare no conflicts of interest.

## Supporting information




**Supporting File**: advs75461‐sup‐0001‐SuppMat.docx.

## Data Availability

The data that support the findings of this study are available from the corresponding author upon reasonable request.
