# Supplementary material for: Development of a Human 3D Immune‐Competent Neurovascular Model Enabling Time‐Resolved Monitoring of Neuroinflammatory Dynamics and Neuroimmune Interactions
Source: Adv Sci (Weinh). 2026 Apr 29;13(41):e75461. doi: 10.1002/advs.75461 (PMC13335724; doi:10.1002/advs.75461)
Supplement: Supplementary file 1 — Supporting File: advs75461‐sup‐0001‐SuppMat.docx. [file ADVS-13-e75461-s001.docx]

Supporting Information

**Development of a Human 3D Immune-Competent Neurovascular Model Enabling Time-Resolved Monitoring of Neuroinflammatory Dynamics and Neuroimmune Interactions**

Pavlo Gordiichuk, Jing Bai, Olurotimi A Bolonduro, Andrew M Silverman, Robert Madison Green, Jason H Lasser, Paul W Fleming, Lishomwa C Ndhlovu, James F Saunders, Dmitry Shvartsman


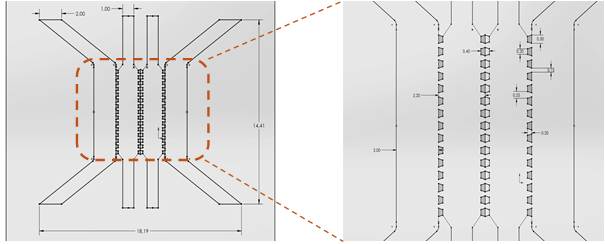


**Figure S1.** CAD drawings correspond to internal channels within the PDMS microfluidic.


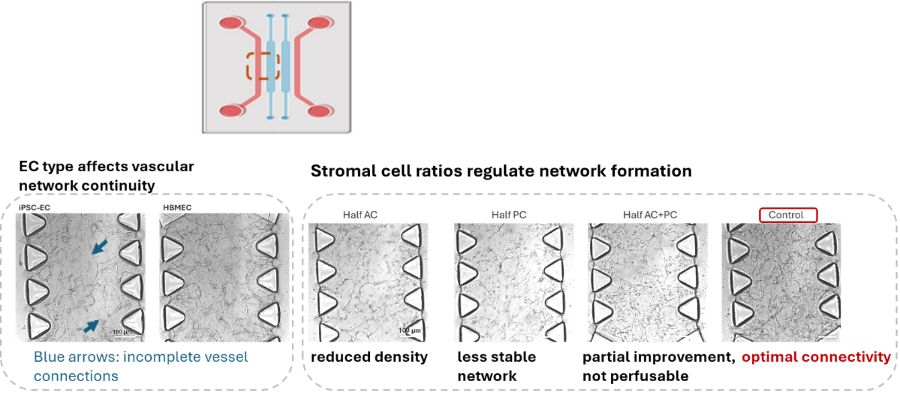


**Figure S2.** Optimization of vascular network formation in the BBB model.

(left) Phase contrast images of vascular networks formed on day 7 using iPSC-derived ECs and HBMECs. Blue arrows indicate regions with incomplete vascular connections, highlighting reduced network continuity in iPSC-EC conditions compared to HBMECs. (right) Phase contrast images showing the effect of astrocyte (AC) and pericyte (PC) ratios on vascular network formation. Reduced AC or PC conditions resulted in decreased vessel continuity or density, whereas the control condition (balanced AC and PC ratio) produced more interconnected and stable vascular networks. Based on these observations, the control condition was selected for subsequent experiments.


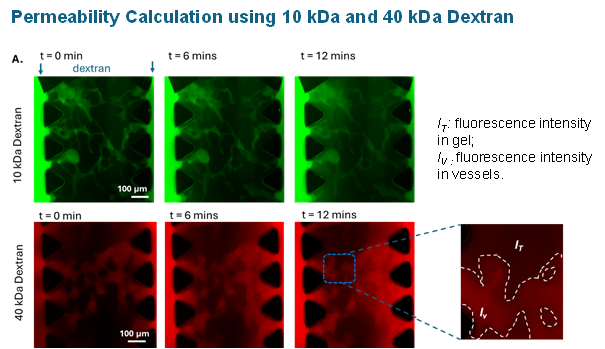


**Figure S3.** Dextran permeability analysis in 3D microfluidic BBB model. A. Representative fluorescence images showing diffusion of 10 kDa FITC-dextran (green) and 40 kDa Texas red-dextran (red) from media channels into the central gel channel over time. Arrows indicate the direction and reservoir through which dextran was applied during the permeability assay. The enlarged region: an example of a cropped image of 200 x 200 µm. IT is the fluorescent intensity of extravascular regions, and IV is the fluorescent intensity of intravascular regions. B. Quantification of permeability for 10 kDa and 40 kDa dextrans. Data are presented as mean ± SEM (n = 7).


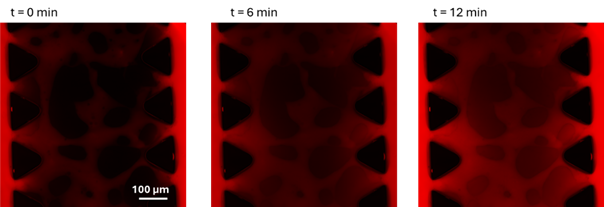


**Figure S4. Zoom into** 40 kDa Texas red-dextran (red) from media channels into the central gel channel over time.


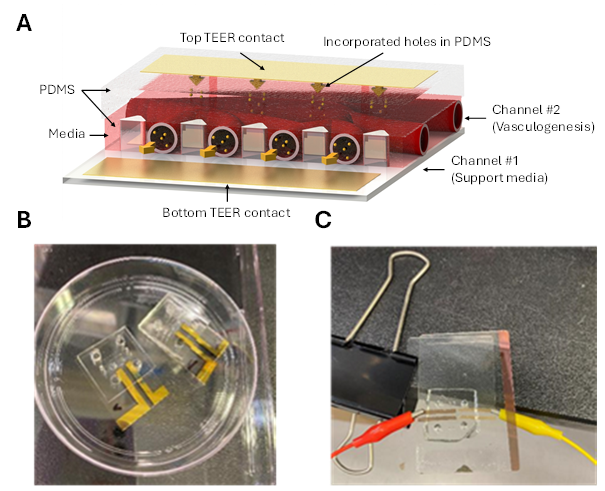


**Figure S5.** (A) Demonstration of TEER electrode positioning relative to each other to facilitate electrical transport between channels #1 and #2 through vascular structures. The 3D image illustrates electronic transport, represented by the yellow sphere indicated with arrows. Other cells have been removed for clarity. (B) Optimized Cr/Au (5 nm/100 nm) electrode structures designed for easier and more stable assembly with microfluidic chips. (C) TEER measurements were performed on the microfluidic chip using two electrodes, with the chip mechanically fixed to the table for stability.


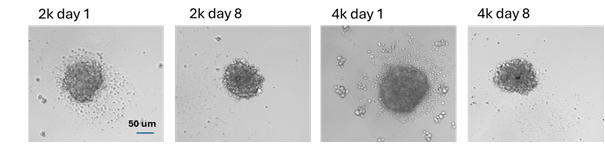


**Figure S6.** Brightfield images of neuron spheroids formed under different seeding densities.

**Figure S7.** Immunofluorescence characterization of NPC spheroids. Representative images of NPC spheroids stained for DAPI (nuclei, blue), PAX6 (neural progenitor marker, red), and Nestin (intermediate filament protein, green).


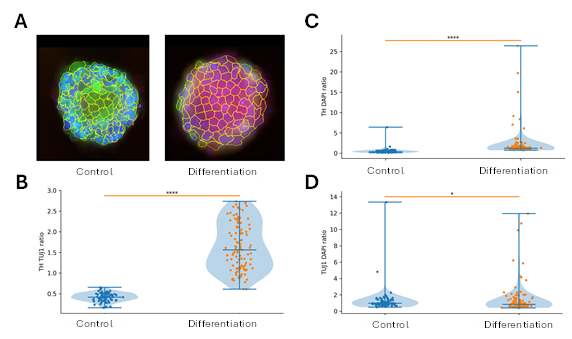


**Figure S8.** (A) Representative immunofluorescence images of organoids stained for DAPI (blue), tyrosine hydroxylase (TH, red), and TUJ1 (green) under two conditions (Control and Differentiation). Organoids were segmented into pseudo-regions using superpixel-based clustering constrained by organoid masks, and fluorescence intensities were quantified within each region. Violin plots show the distribution of TH/TUJ1 (B), TH/DAPI (C), and TUJ1/DAPI (D) ratios across pseudo-regions, with individual data points overlaid. Statistical significance was assessed using a two-sided Mann–Whitney U test (*p < 0.05, **p < 0.01, ***p < 0.001, ****p < 0.0001). Increased TH-associated signal relative to TUJ1 and DAPI indicates enhanced dopaminergic differentiation in Differentiation compared to Control, without a corresponding increase in overall cell density.


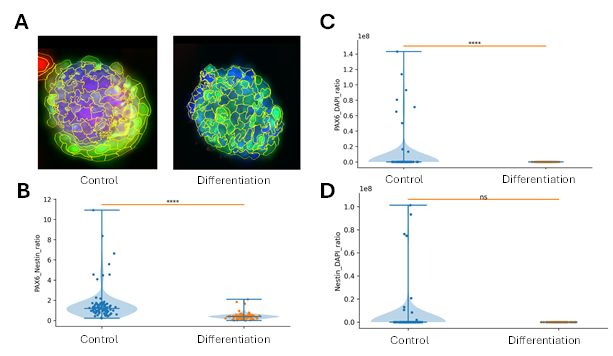


**Figure S9.** (A) Representative immunofluorescence images of organoids stained for DAPI (blue), PAX6 (red), and Nestin (green) under two conditions (Control and Differentiation). Organoids were segmented into pseudo-regions using superpixel-based clustering constrained by organoid masks, and fluorescence intensities were quantified within each region. Violin plots show the distribution of PAX6/Nestin (B), PAX6/DAPI (C), and Nestin/DAPI (D) ratios across pseudo-regions, with individual data points overlaid. Statistical significance was assessed using a two-sided Mann–Whitney U test (*p < 0.05, **p < 0.01, ***p < 0.001, ****p < 0.0001). Increased PAX6-associated signal relative to Nestin and DAPI indicates enhanced neural progenitor specification in Differentiation compared to Controls. (ns: not significant)

**Figure S10.** (top panel) Viability assessment of microglial spheroids under suspension culture conditions. A. Brightfield image showing the morphology of microglial spheroids formed in low-adhesion wells. B. Live/dead assay performed 48 hours after suspension using Calcein-AM (green, live cells) and PI (red, dead cells). (bottom panel) Microglia markers, IF staining in suspension culture.

**Figure S11**. Astrocytes or astrocyte-conditioned media were applied to Side B, resulting in localized blockage of vessel openings. A. Position of different cell types, astrocyte and Astrocyte‑conditioned media (ACM), and image showing that astrocytes “block” vessel opening. B. Confocal images show intact vascular networks in the central chamber with sealed openings on Side B (blue arrows indicate the position of astrocytes/ACM). C. Live cells images of Astrocytes in gel. Astrocyte‑conditioned media was used to expose endothelial cells to astrocyte‑secreted factors, which have been shown to modulate endothelial barrier properties and paracrine signalling *in vitro*, influencing tight junction expression and vascular behaviour in engineered vascular models ^[95]^ .

The method illustrates the workflow for generating a BBB model with only one opening, designed to mimic selective transport from the vascular lumen into the brain parenchyma. In this configuration, Side A contains the only perfusable outlet, whereas Side B is designated as the brain-facing region where vessel openings are intentionally sealed. To block the vessel openings on Side B, astrocytes were incorporated on the parenchymal side using two approaches. In the cell-seeding condition, astrocytes were mixed into a fibrin gel at 2 × 10^6^ cells/mL and allowed to attach at the vessel interface at day 5 (BBB culture, all the other procedures remain the same), before vessel openings. Basically, astrocytes were first mixed with fibrin and introduced through one inlet of the neuron channel. Before the fibrin began to gel, the mixture was quickly withdrawn from the opposite inlet, allowing astrocytes to remain only along the targeted side of the channel while leaving the central neuron-gel region empty. After a 20-minute gelation period, endothelial cell (EC) media was added to the same channel to support cell attachment and stabilization.

In the ACM (astrocyte-conditioned media) condition, media were collected from 1 × 10⁶ astrocytes cultured in 3 mL endothelial base medium for 48 h, and the conditioned media were applied to Side B. Both treatments effectively “sealed” the Side B vessel openings, as reflected by reduced dye penetration and the absence of perfusion channels by confocal imaging, while preserving robust perfusion on Side A.

This sealing effect is consistent with known astrocyte-mediated angiostatic signaling—particularly TGF-β and other inhibitory factors —which suppress endothelial tip-cell extension and limit sprouting angiogenesis. Leveraging this mechanism enables the model to establish a physiologically relevant brain-facing barrier, requiring solutes to traverse the BBB before reaching the parenchymal compartment.


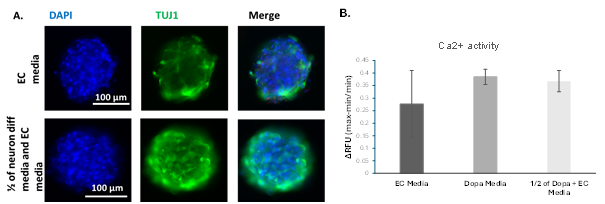


**Figure S12**. A. The panel shows immunofluorescence staining of spheroids cultured in endothelial cell (EC) media (top row) versus dopaminergic neuronal media (bottom row). Staining for DAPI (nuclei, blue) and TUJ1 (neuronal marker, green) confirmed neuronal identity in both conditions. However, TUJ1 intensity appeared higher in the dopaminergic media condition, suggesting enhanced neuronal differentiation or maintenance. B. Ca^2+^ measurements on the corresponding samples.


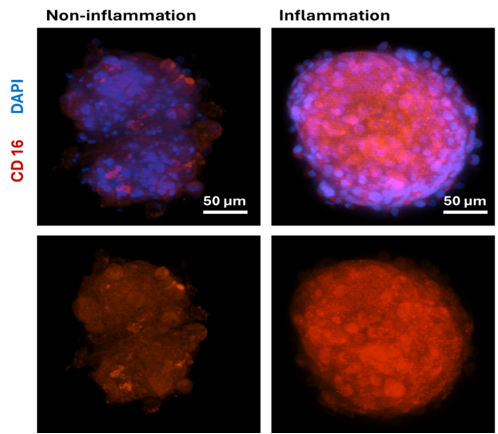


**Figure S13.** CD16 expression as a marker of microglial activation under inflammatory conditions Those experiments confirm that LPS activates microglia pro-inflammatory conditions in our MPS model seen in TEER measurements.


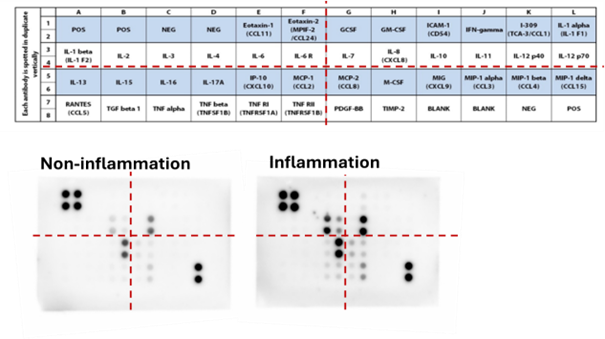


**Figure S14**. Upper panels show the reference array map listing the 42 cytokines included in the panel. Lowel panels display representative chemiluminescent blot images from control and LPS-treated samples.


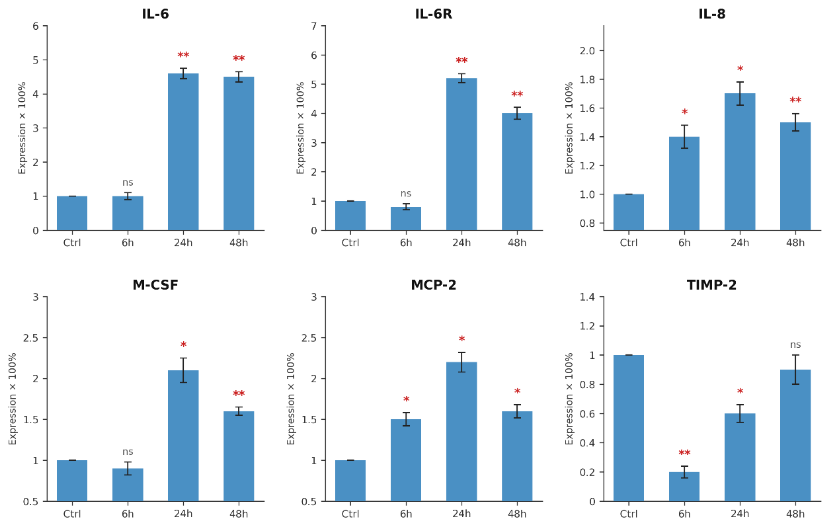


**Figure S15A.** Cytokine array analysis of six common inflammatory cytokines in response to TNF-α. An one-sample t-test was performed for each time point against a theoretical mean of 1 (normalized control), with n = 3 biological replicates, significance thresholds were set at * p < 0.05 and ** p < 0.01.


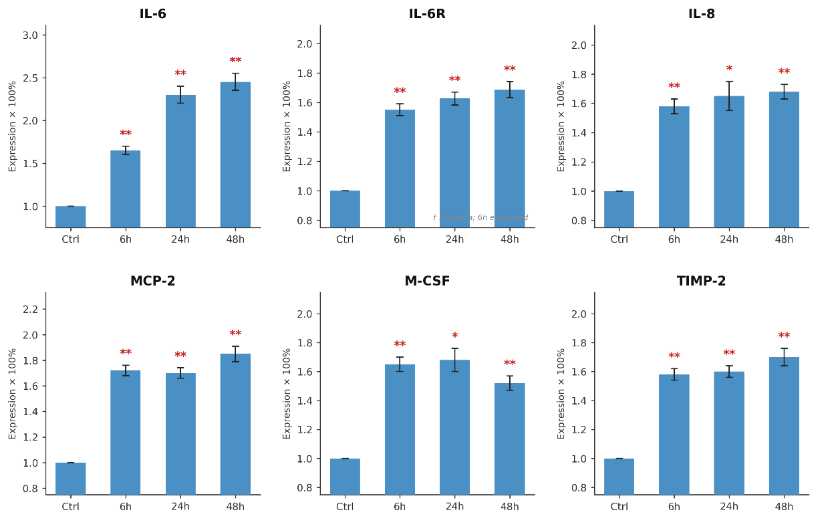


**Figure S15B.** Cytokine array analysis of six common inflammatory cytokines in response to LPS. An one-sample t-test was performed for each time point against a theoretical mean of 1 (normalized control), with n = 3 biological replicates, significance thresholds were set at * p < 0.05 and ** p < 0.01.

**Figure S16.** Time-course analysis of inflammatory responses in the microfluidic neurovascular model measured using TEER sensors. (A, D) Time-dependent TEER measurements of three 3D BBB devices following exposure to LPS (1 µg/mL). (B, E) Time-dependent TEER measurements of three 3D BBB devices following exposure to TNF-α (10 ng/mL). (C, F) Time-dependent TEER measurements of three 3D BBB devices used as controls, without exposure to inflammatory agents, measured under the same conditions as the treated samples. The collected measurements were used to determine mean values and their corresponding standard deviations.

**

**Figure S17.** (A) TEER time courses for Control, LPS, and TNF-α over 48 h (mean ± SD, n = 3). LPS induces a delayed but pronounced decrease in barrier integrity, with significant differences relative to Control at 7–10 h (* to **), whereas TNF-α produces an earlier but moderate decrease (6–9 h, *). Holm-corrected omnibus comparisons at each time point are indicated above the curves (ns, *, **, ****). (B) TEER normalized to baseline (0 h), expressed as % baseline, highlighting differences in relative disruption magnitude and recovery kinetics. (C) Peak disruption amplitude (maximum ΔTEER from baseline). LPS shows a significantly greater decrease compared to Control (**, p ≈ 0.008) and TNF-α (*, p ≈ 0.02), while TNF-α does not significantly differ from Control (ns, p ≈ 0.18). (D) Time to minimum TEER. LPS reaches maximal disruption significantly later than Control (**, p ≈ 0.006) and TNF-α (*, p ≈ 0.03), consistent with delayed barrier breakdown kinetics. Statistical analysis: TEER trajectories were analyzed using a linear mixed-effects model with treatment, time, and their interaction as fixed effects and replicate identity as a random effect to account for repeated measurements. Models were fitted using maximum likelihood, and statistical significance was assessed via likelihood-ratio tests comparing nested models. This analysis revealed significant effects of time, treatment, and a strong time × treatment interaction, demonstrating distinct temporal response profiles. Timepoint-specific comparisons were performed using one-way ANOVA with Holm correction for multiple comparisons, and pairwise group differences were evaluated using Welch’s t-test with Holm correction.
